# Skeletal progenitor LRP1 deficiency causes severe and persistent skeletal defects with Wnt pathway dysregulation

**DOI:** 10.1038/s41413-024-00393-x

**Published:** 2025-01-26

**Authors:** Mohammad Alhashmi, Abdulrahman M. E. Gremida, Santosh K. Maharana, Marco Antonaci, Amy Kerr, Shijian Fu, Sharna Lunn, David A. Turner, Noor A. Al-Maslamani, Ke Liu, Maria M. Meschis, Hazel Sutherland, Peter Wilson, Peter Clegg, Grant N. Wheeler, Robert J. van ‘t Hof, George Bou-Gharios, Kazuhiro Yamamoto

**Affiliations:** 1 Institute of Life Course and Medical Sciences, Faculty of Health and Life Sciences, University of Liverpool, Liverpool, UK; 2 School of Biological Sciences, University of East Anglia, Norwich Research Park, Norwich, Norfolk UK; 3 Present Address: Department of Medical Laboratory Sciences, Faculty of Applied Medical Sciences, King Abdulaziz University, Jeddah, Saudi Arabia; 4 Present Address: King Fahd Medical Research Center, King Abdulaziz University, Jeddah, Saudi Arabia; 5 Present Address: VANTHOF SCIENTIFIC, Torun, Poland

**Keywords:** Physiology, Pathogenesis

## Abstract

Low-density lipoprotein receptor-related protein 1 (LRP1) is a multifunctional endocytic receptor whose dysfunction is linked to developmental dysplasia of the hip, osteoporosis and osteoarthritis. Our work addresses the critical question of how these skeletal pathologies emerge. Here, we show the abundant expression of LRP1 in skeletal progenitor cells at mouse embryonic stage E10.5 and onwards, especially in the perichondrium, the stem cell layer surrounding developing limbs essential for bone formation. *Lrp1* deficiency in these stem cells causes joint fusion, malformation of cartilage/bone template and markedly delayed or lack of primary ossification. These abnormalities, which resemble phenotypes associated with Wnt signalling pathways, result in severe and persistent skeletal defects including a severe deficit in hip joint and patella, and markedly deformed and low-density long bones leading to dwarfism and impaired mobility. Mechanistically, we show that LRP1 regulates core non-canonical Wnt/planar cell polarity (PCP) components that may explain the malformation of long bones. LRP1 directly binds to Wnt5a, facilitates its cell-association and endocytic degradation and recycling. In the developing limbs, LRP1 partially colocalises with Wnt5a and its deficiency alters abundance and distribution of Wnt5a and Vangl2. Finally, using *Xenopus* as a model system, we show the regulatory role for LRP1 in Wnt/PCP signalling. We propose that in skeletal progenitors, LRP1 plays a critical role in formation and maturity of multiple bones and joints by regulating Wnt signalling, providing novel insights into the fundamental processes of morphogenesis and the emergence of skeletal pathologies.

## Introduction

The low-density lipoprotein (LDL) receptor-related protein 1 (LRP1) is widely expressed type 1 transmembrane protein in adult tissues^1,2^ that regulates cellular events by modulating the levels of structurally and functionally diverse extracellular molecules via clathrin-dependent endocytosis.^3,4^ Our previous studies demonstrated that LRP1 plays an important role in the turnover of extracellular matrix (ECM) components in articular cartilage by mediating endocytic clearance of cartilage-degrading proteinases and their inhibitors.^5–10^ This endocytic process is impaired in cartilage under inflammatory conditions or in osteoarthritis (OA), the most prevalent age-related degenerative joint disease.^11^ LRP1 also participates in signalling pathways through interaction with membrane receptors and cytoplasmic adaptor proteins. The cytoplasmic NPxY motifs within the LRP1 intracellular domain also provide binding sites for a set of signalling proteins.^12–14^

Global deletion of the *Lrp1* gene in mice results in embryonic lethality at E13.5.^15,16^ The homozygous and heterozygous *Lrp1* null^tm1.1(KOMP)Wtsi^ mice exhibit multiple phenotypes, as described in the Mouse Genome Informatics database (MGI: 5495233). Tissue-specific *Lrp1* deletion in mice has revealed various biological roles of LRP1 including lipoprotein metabolism,^17,18^ insulin signalling,^19^ inflammation,^20,21^ heart development,^22^ vascular wall integrity and remodelling^23–26^ and bone development and remodelling.^27–30^ Together, these studies highlight a critical and non-redundant role of LRP1 in development as well as adult tissue homoeostasis.

*LRP1* single nucleotide polymorphisms are associated with a decrease in bone mineral density and content.^31^ A recent study by Yan et al. ^32^ identified mutations in *LRP1* including R1783W with developmental dysplasia of the hip (DDH) patients. In mice, mutant *Lrp1*^*R1783W*^ homozygote and heterozygote mice exhibited delayed Y-shaped triradiate cartilage and smaller acetabulum in 8-week and 16-week-old mice, respectively.^32^ Heterozygous global *Lrp1* knockout (KO) mice also developed a hip dysplasia phenotype. In contrast, a bone and cartilage conditional KO mice (*Lrp1*^flox/flox^*/Col2a1*^Cre^) showed shortened bones and cartilage growth plate.^27^ In vitro, siRNA-mediated Lrp1 gene-silencing reduced chondrogenesis of human mesenchymal stem cells.^33^ These studies suggest that function of LRP1 during synovial joint and bone formation is cell type- and time-dependent. However, LRP1 expression and distribution in the developmental stages remains incompletely understood.

In this study, we addressed the critical questions of when and where LRP1 is expressed during skeletal development of the mouse, how deficiency of LRP1 leads to skeletal pathologies and how long it persists, and which molecular mechanisms underpin the defects. We showed that LRP1 is abundantly expressed in skeletal progenitor cells at embryonic stage E10.5 and onwards, in particular in the perichondrium.^34^ To investigate the role of skeletal progenitor LRP1, we generated a conditional KO of *Lrp1* using the enhancer of paired-related homebox gene-1 (*Prrx1*), which is expressed in the early limb bud mesenchyme and a subset of craniofacial mesenchyme.^35,36^ *Lrp1*^flox/flox^*/Prrx1*^Cre^ mice exhibited severe and persistent malformation of multiple bones and synovial joints, which were not evident in either the *Lrp1*^*R1783W*^ or *Lrp1*^flox/flox^*/Col2a1*^Cre^ mice. Our exploration of molecular mechanisms showed unique regulation of non-canonical Wnt/planar cell polarity (PCP) components and signalling by LRP1. We propose that LRP1 plays a critical role in skeletal development by partly regulating Wnt signalling, which governs a myriad of biological processes underlying the development and maintenance of adult tissue homoeostasis.

## Results

### LRP1 is abundantly expressed in skeletal progenitor cells, in particular in the perichondrium

**Fig. 1:**
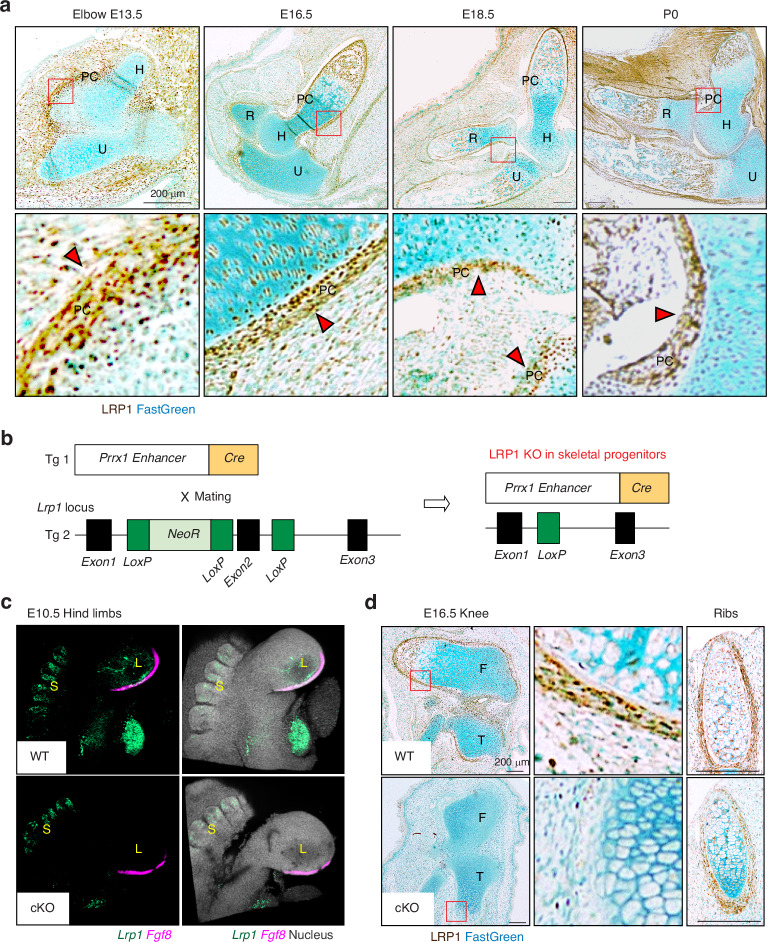
LRP1 is abundantly expressed in skeletal progenitor cells, in particular in the perichondrium. **a** Representative images of immunohistochemical staining of LRP1 and fast green counterstaining in WT E13.5-P0 elbow joint sections of *Lrp1*^flox/flox^ mice. Scale bar, 200 µm. H, humerus; R, radius; U, ulna; PC, perichondrium. Regions delineated by the red squares in the panels have been magnified in the lower panels. Arrow heads indicate abundant LRP1 expression in perichondrium layers. **b** Schematic diagram showing the constructs used to generate skeletal progenitor-selective LRP1 conditional knockout mice. Transgenic mouse lines harbouring *Prrx1* limb enhancer Cre (Tg 1) and floxed *Lrp1* (Tg 2) were used to establish the *Lrp1*^flox/flox^*/Prrx1*^Cre^ line. **c** In situ hybridisation chain reaction for *Lrp1* and *Fgf8* mRNA expression in E10.5 hind limbs of WT and *Lrp1*^flox/flox^*/Prrx1*^Cre^ homozygote conditional KO (cKO) mice. *Lrp1* and *Fgf8* were visualised as described under “Materials and Methods”. L, bulging limb bud; S, somite. **d** Representative images of immunohistochemical staining of LRP1 and fast green counterstaining in E16.5 knee and rib sections of WT and *Lrp1*^flox/flox^*/Prrx1*^Cre^ homozygote conditional KO (cKO) mice. Regions delineated by the red squares in the upper panels have been magnified in the lower panels. Scale bar, 200 µm. F, femur; T, tibia

We investigated the distribution of LRP1 during skeletal development, which had thus far remained unknown. Histological investigation of LRP1 protein in developing limbs showed that LRP1 is abundantly expressed in E13.5- to newborn (P0) elbow joints, with the strongest immunosignals of LRP1 detected in the perichondrium layers, a dense stem cell layer surrounding developing limbs essential for bone formation (Fig. 1a). To investigate the role of skeletal progenitor LRP1 in skeletal development in vivo, we have established a double transgenic mouse line *Lrp1*^flox/flox^*/Prrx1*^Cre^ (Fig. 1b). In this strain, a 2.4 kb *Prrx1* enhancer directs the transgene expression in undifferentiated mesenchyme in the developing limb buds around embryonic day 9.5 (E9.5).^35–38^ Analysis of the *Lrp1* transcript by in situ hybridisation chain reaction (HCR)^39^ in E10.5 wild-type (WT) embryos (*Lrp1*^flox/flox^) showed its expression in bulging limb bud mesenchyme and somite (Fig. 1c). *Lrp1* expression was specifically diminished in limb buds but not in somite of E10.5 *Lrp1*^flox/flox^*/Prrx1*^Cre^ embryos. The expression of *Fgf8*, which is present in the apical ectodermal ridge of limb buds, was unaltered in *Lrp1* mutant embryos (Fig. 1c). Immunohistochemistry of LRP1 further confirmed specific deletion of LRP1 in *Prrx1* expressing skeletal progenitor cells in the developing limbs in E16.5 knee but not in their ribs (Fig. 1d).

### Conditional deletion of *Lrp1* in skeletal progenitors impairs early bone and joint formation

**Fig. 2:**
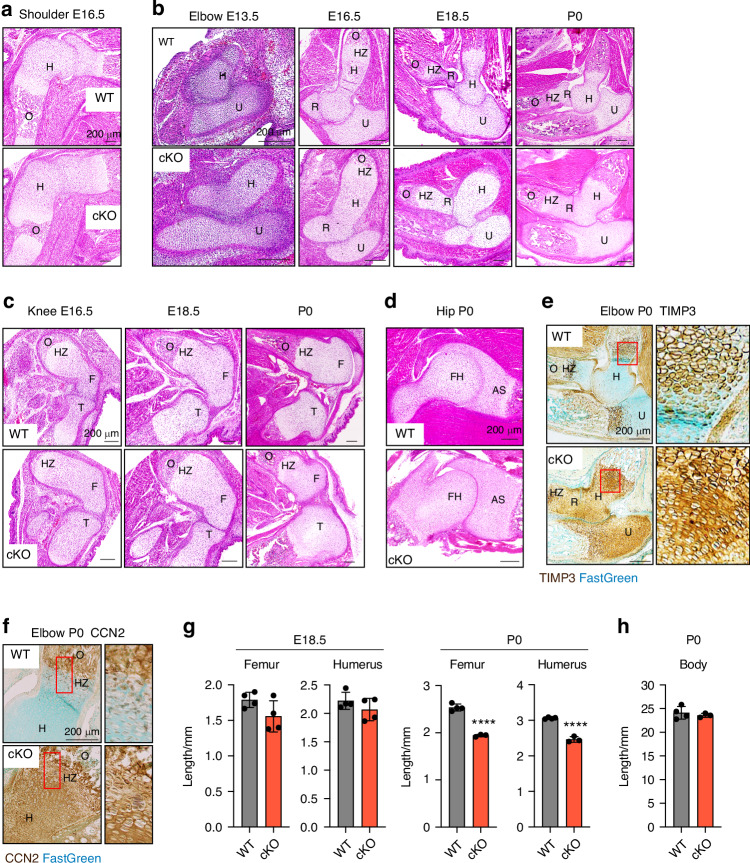
Conditional deletion of *Lrp1* in skeletal progenitors impairs early bone and joint formation. Representative images of H&E staining of E16.5 shoulder (**a**), E13.5-P0 elbow (**b**), E16.5-P0 knee (**c**) and P0 hip (**d**) sections of WT and *Lrp1*^flox/flox^*/Prrx1*^Cre^ homozygote conditional KO (cKO) mice. Scale bar, 200 µm. H, humerus; R, radius; U, ulna; HZ, hypertrophic zone; O, primary ossification centre; F, femur; FH, femur head; T, tibia; AS, acetabulum socket. **e** and **f**, Representative images of immunohistochemical staining of TIMP3 (**e**) and CCN2 (**e**) and fast green counterstaining in E16.5 knee sections of WT and cKO mice. Regions delineated by the red squares in the panels have been magnified in the right panels. Scale bar, 200 µm. **g** and **h**, Length of femur and humerus mineralised bone (**g**) and body (**h**) of E18.5 (**g**) and P0 (gand **h**) WT and cKO mice. Circles represent individual mice and bars show the mean ± *SD*. *P* values were evaluated by 2-tailed Student’s t test. ****P < 0.000 1

Haematoxylin and eosin (H&E) staining of limbs at different embryonic stages revealed fusion of joints, malformation of cartilage/bone template and markedly delayed or lack of primary ossification in E16.5 *Lrp1*^flox/flox^*/Prrx1*^Cre^ homozygote shoulder, elbow and knee joints (Fig. 2a–c) and the defects become more severe in E18.5 and P0 neonates (Fig. 2b, c). Striking joint malformations were observed in P0 *Lrp1*^flox/flox^*/Prrx1*^Cre^ hip joints (Fig. 2d). These defects were observed in all homozygote mice analysed in this study but not in heterozygote (*Lrp1*^flox/wt^*/Prrx1*^Cre^) littermates. Histological investigation of LRP1 ligands including tissue inhibitor of matrix metalloproteinase 3 (TIMP3) (Fig. 2e) and CCN2 (Fig. 2f) revealed their aberrant accumulation in P0 *Lrp1*^flox/flox^*/Prrx1*^Cre^ compared with limbs of WT littermates (*Lrp1*^flox/flox^ or *Lrp1*^wt/wt^*/Prrx1*^Cre^), indicating a loss of LRP1 function and its critical role in their tissue availability. High resolution µCT scanning showed that length of femur and humerus mineralised bone was shorter in P0 *Lrp1*^flox/flox^*/Prrx1*^Cre^ compared with WT mice, whereas no significant difference was observed in E18.5 embryos (Fig. 2g). No significant differences in body length were observed in P0 neonates (Fig. 2h).

These joint and cartilage/bone template defects have not been reported for the *Lrp1*^*R1783W*^ ^32^ or *Lrp1*^flox/flox^*/Col2a1*^Cre^ mice^27^ most likely because they reflect events that occurred earlier than E12.5 when the transgene expression in chondrocytes is driven by *Col2a1* regulatory regions.^40^ To further evaluate the role of chondrocyte LRP1 in the cKO early skeletal phenotype, we generated a double transgenic mouse line *Lrp1*^flox/flox^*/Acan*^CreERT2^.^41^ In contrast to *Prrx1* expression,^36,37^ *Aggrecan* expression was detected in the E13.0 forelimb and in proliferating and hypertrophic chondrocytes in ulna and radius but not in perichondrium by E15.5.^41^ *Lrp1* was deleted at the different embryonic stages and E19.5 embryos were examined (Fig. S1). *Lrp1* gene excision was confirmed by *Lrp1 exon 2* PCR (Fig. S1B) but histological analysis and bone length measurement confirmed normal skeletal development in E19.5 *Lrp1*^flox/flox^*/Acan*^CreERT2^ mice (Fig. S1C–F). Histological investigation of TIMP3 showed its accumulation in E19.5 *Lrp1*^flox/flox^*/Acan*^CreERT2^ limbs compared with WT littermates (Fig. S1G) but the difference is much less than that in E16.5 *Lrp1*^flox/flox^*/Prrx1*^Cre^ limbs.

### LRP1 deficiency in skeletal progenitors results in dwarfism, impaired mobility, abnormal gaits and fore/hind limb malformation

**Fig. 3:**
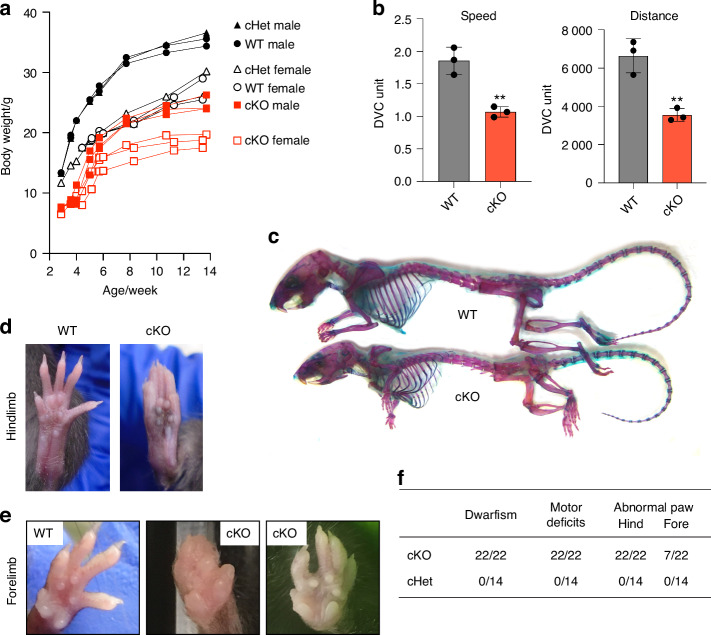
LRP1 deficiency in skeletal progenitors results in dwarfism, impaired mobility and fore/hind limb malformation. **a** Mouse weight measurement from 3-14 weeks after birth. Closed (male) and open (female) black circles (WT), black triangles (cHet; *Lrp1*^flox/wt^*/Prrx1*^Cre^ heterozygote conditional KO), and red squares (cKO; *Lrp1*^flox/flox^*/Prrx1*^Cre^ homozygote conditional KO) represent individual mice. **b** Total distance and average speed of WT and cKO mice. Locomotor activity of 6-week-old mice measured by the continuous automated home-cage monitoring system for 4 weeks. Circles represent individual mice and bars show the mean ± *SD*. **P < 0.01 by 2-tailed Student’s t test. **c** Whole-mount skeletal staining of 14-week-old mice with Alcian blue and Alizarin red. **d** Photographs of 8-week-old WT and the cKO hind paws. **e** Photographs of 8-14-week-old WT and the cKO fore paws. **f** Table showing the frequencies of the identified phenotypes

We next investigated the impact of early skeletal developmental defects in postnatal *Lrp1*^flox/flox^*/Prrx1*^Cre^ mice. The mice survived into adulthood, but the weight of the homozygote mice was consistently lower than their WT and heterozygote littermates starting 3 weeks up to 14 weeks after birth (Fig. 3a). Strikingly, *Lrp1*^flox/flox^*/Prrx1*^Cre^ mice consistently showed altered posture and impaired mobility at birth which was persistent up until 14 weeks of age (Movies S1 and S2). A continuous automated home-cage monitoring system for a 4-week period from week 6 showed significantly reduced locomotor activity of *Lrp1*^flox/flox^*/Prrx1*^Cre^ mice compared with WT littermates (Fig. 3b). Whole-mount skeletal staining of 14-week-old postnatal adult mice with Alcian blue and Alizarin red showed that *Lrp1*^flox/flox^*/Prrx1*^Cre^ mice had shorter limbs and smaller stature than their WT littermates (Fig. 3c). *Lrp1*^flox/flox^*/Prrx1*^Cre^ mice were unable to open digits in their hind limb (Fig. 3d). As an occasional feature in *Lrp1*^flox/flox^*/Prrx1*^Cre^ mice, fused and abnormal digits were observed in their fore limb (Fig. 3e, f). None of these phenotypes were observed in *Lrp1*^flox/flox^*/Prrx1*^Cre^ heterozygote mice, suggesting an autosomal recessive phenotype.

### Severe and persistent defects in multiple bones and joints in *Lrp1*^flox/flox^*/Prrx1*^Cre^ mice

**Fig. 4:**
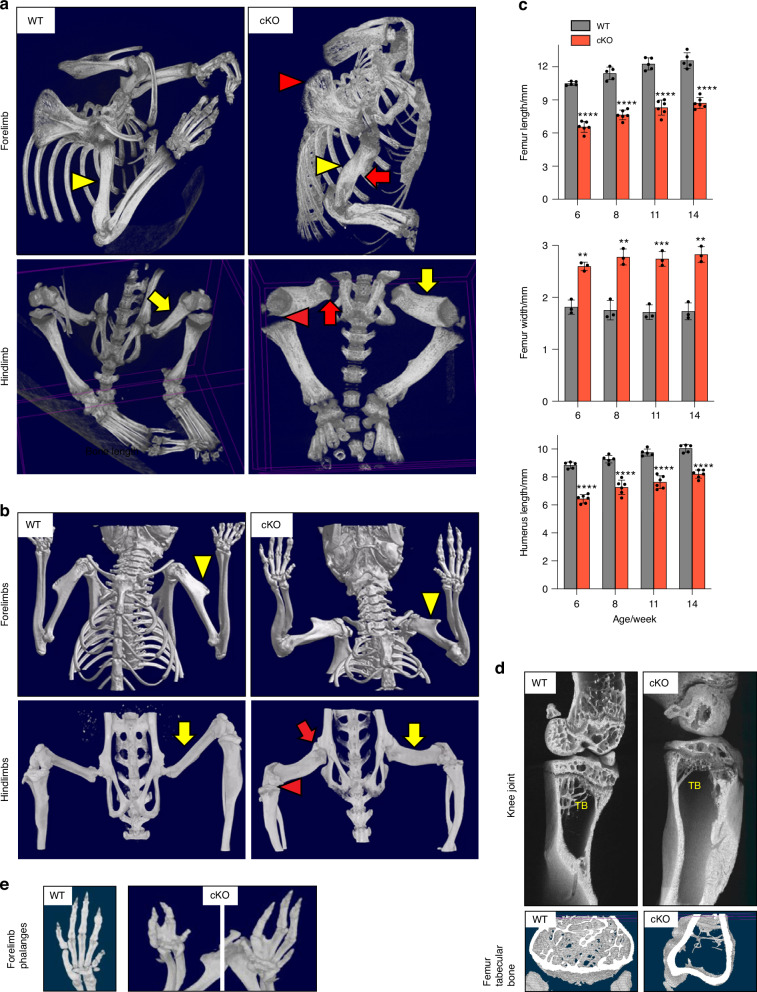
Severe and persistent defects in multiple bones and joint in *Lrp1*^flox/flox^*/Prrx1*^Cre^ mice. Representative images of in vivo X-ray analysis of limbs of 2-week-old (**a**) and 14-week-old (**b**) WT and *Lrp1*^flox/flox^*/Prrx1*^Cre^ homozygote conditional KO (cKO) mice. Yellow arrowheads and arrows indicate humerus and femur quantified in **c**. Red arrowheads and arrows indicate undefined shoulder blades and twisted long bones (top panels), and undefined knee and lack of hip joints (bottom panels) in cKO mice, respectively. **c** Femur and humerus bone length and femur width of 6-14-week-old mice. Circles represent individual mice and bars show the mean ± *SD*. **P < 0.01; ***P < 0.001; ****P < 0.000 1 by 2-tailed Student’s *t* test. **d** Representative images of high-resolution μCT analysis of femur trabecular bones of 14-week-old mice. TB, trabecular bone. **e** Representative images of in vivo X-ray analysis of forelimb phalanges of 14-week-old cKO mice

In vivo micro-computed tomography (μCT) scanning of 2-week-old *Lrp1*^flox/flox^*/Prrx1*^Cre^ mice revealed that long bones were not only shorter but markedly thicker and twisted, with much delayed or a lack of emergence of secondary ossification centres (Fig. 4a). The upper limbs were abnormally twisted and the scapular were poorly defined compared with WT. The absence of crescent-shaped acetabulum sockets, rounded femoral heads and patella bone in *Lrp1*^flox/flox^*/Prrx1*^Cre^ mice suggested an impaired mobility. Notably, these bone and joint defects remained defective in 14-week-old mice (Fig. 4b). The observed difference in the length and width of femur and humerus bones between 6-week-old WT and *Lrp1*^flox/flox^*/Prrx1*^Cre^ mice persisted in 14-week-old mice (Fig. 4c and S2). High-resolution µCT scanning of a 14-week-old *Lrp1*^flox/flox^*/Prrx1*^Cre^ tibia revealed a substantial reduction in trabecular bone density with virtual absence of primary spongiosa compared to the WT tibia (Fig. 4d). Fusion and deformities of phalanges were observed in *Lrp1*^flox/flox^*/Prrx1*^Cre^ forelimb with fused and abnormal digits (Fig. 4e).

### Defects in growth plate, organisation of columnar chondrocytes, secondary ossification, articulation and cavitation of joints and proteoglycan turnover in *Lrp1*^flox/flox^*/Prrx1*^Cre^ mice

**Fig. 5:**
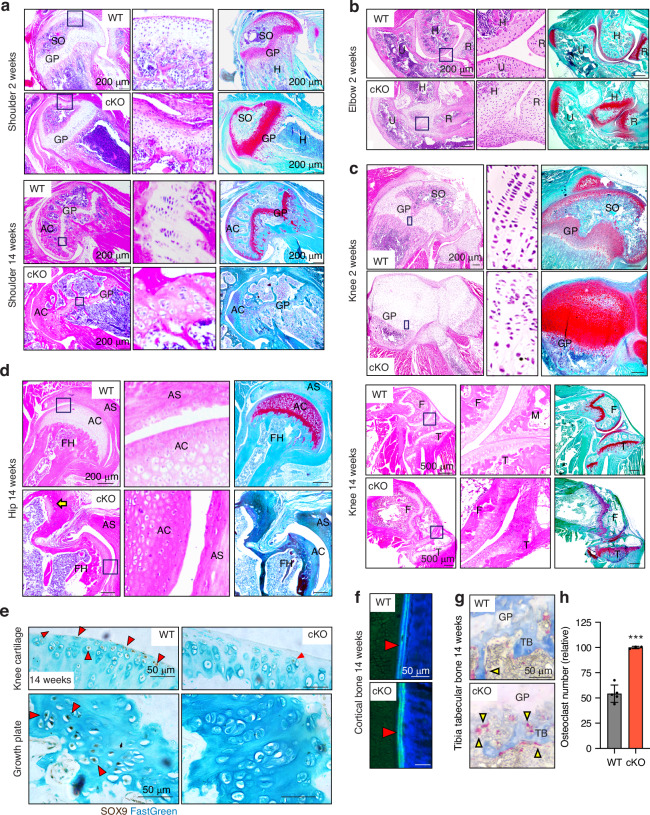
Defects in growth plate, organisation of columnar chondrocytes, secondary ossification, articulation and cavitation of joints and proteoglycan turnover in *Lrp1*^flox/flox^*/Prrx1*^Cre^ mice. Representative images of H&E and safranin-O/fast green staining in shoulder (**a**), elbow (**b**), knee (**c**) and hip (**d**) joint sections of 2- and/or 14-week-old WT and *Lrp1*^flox/flox^*/Prrx1*^Cre^ **(**cKO) mice. Arrow indicates the extra groove of femoral heads (**d**). Regions delineated by the dark blue squares in the panels have been magnified in the right panels. Scale bar, 200 µm. GP, growth plate; AC, articular cartilage; F, femur; T, tibia; M, menisci. **e**, Representative images of immunohistochemical staining of SOX9 in knee articular cartilage and growth plate sections of 14-week-old WT and cKO mice. Arrow heads indicate cells with SOX9 positive staining. Scale bar, 50 µm. **f** Representative images of fluorescent microscopy analysis of calcein-double stained cortical bones in 14-week-old mice (*n* = 3 each). Red arrowheads indicate calcein double staining in cortical bone. Scale bar, 50 µm. **g** TRAP and aniline blue staining of tibia trabecular bone of 14-week-old mice. Yellow arrowheads highlight osteoclast staining. Scale bar, 50 µm. TB, trabecular bone; GP, growth plate. **h** Measurement of osteoclast numbers in WT and *Lrp1*^flox/flox^*/Prrx1*^Cre^ (cKO) trabecular bones. Circles represent individual mice and bars show the mean ± *SD*. ***P < 0.001 by 2-tailed Student’s *t* test

To investigate details of the multiple and severe skeletal defects in *Lrp1*^flox/flox^*/Prrx1*^Cre^ mice, we histologically examined the bone and joint sections. H&E staining of 2-week and/or 14-week-old *Lrp1*^flox/flox^*/Prrx1*^Cre^ shoulder, elbow and knee revealed striking defects (Fig. 5a–c). These included a deformed and disrupted growth plate with poorly stacked columnar chondrocytes, markedly delayed secondary ossification, impaired articulation as a result of fused bone ends and lack of articular cartilage formation. Safranin-O/fast green staining further revealed proteoglycan depletion in articular cartilage and the growth plate in adulthood at 14-week-old mice but not during growth phase in 2-week-old mice (Fig. 5a–c). Similar results were also obtained by Toluidine blue staining. Notably, femoral heads were substantially deformed with an extra groove juxtaposed to a poorly developed acetabulum socket (Fig. 5d). Sox9, a pivotal transcription factor expressed in developing and adult cartilage,^42–44^ was almost absent in the articular cartilage and growth plate of 14-week-old *Lrp1*^flox/flox^*/Prrx1*^Cre^ compared with WT mice (Fig. 5e), emphasising defects in cartilage development.

We then tested dynamic histomorphometry of bone formation using calcein-double labelling in 14-week-old mice and showed a comparable rate of bone formation rate between WT and *Lrp1*^flox/flox^*/Prrx1*^Cre^ mice (Fig. 5f and S3A–C). Further analysis using tartrate-resistant acid phosphatase (TRAP) staining for osteoclasts revealed approximately 1.9-fold increased osteoclast activity in 14-week-old *Lrp1*^flox/flox^*/Prrx1*^Cre^ compared with WT littermates (Fig. 5g, h and S3DE). These results were consistent with *Lrp1* deficiency in osteoblasts^28^ or osteoclasts^29^ resulting in low bone-mass phenotype due to increased osteoclastogenesis.

### LRP1 mediates endocytosis of Wnt5a and Wnt11, core non-canonical Wnt/planar cell polarity (PCP) pathway components

**Fig. 6:**
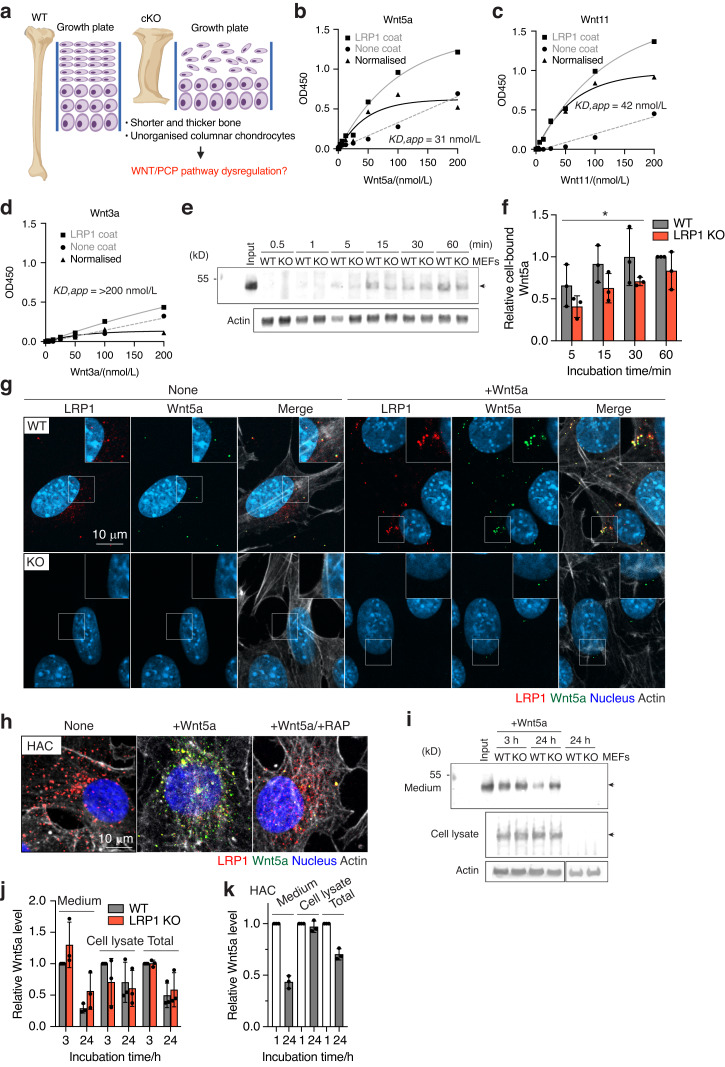
LRP1 mediates endocytosis of Wnt5a, a core non-canonical WNT/planar cell polarity (PCP) pathway component. **a** Schematic diagram illustrating the long bone phenotype of *Lrp1*^*flox/flox*^*/Prrx1*^*Cre*^ mice. Full-length sLRP1 was coated onto microtiter plates and the binding of 0–200 nmol/L Wnt5a (**b**), Wnt11 (**c**), Wnt3a (**d**) was measured using specific antibody for each Wnt as described under “Materials and Methods”. Mean values of technical duplicates for none-coating, LRP1-coating and after normalisation were shown as circles, squares and triangles, respectively. Extrapolated *K*_*D,app*_ values were estimated based on one-phase decay nonlinear fit analysis (black lines). WT and LRP1 KO MEFs (*n* = 3) were incubated with 40 nmol/L Wnt5a for 0.5–60 min and Wnt5a in the cell lysate was detected by Western blotting (**e**). The relative amount of Wnt5a was expressed by taking the amount of Wnt5a after 60-min incubation as 1 (**f**). Circles represent individual mice and bars show the mean ± *S*values for the amount of Wnt5a after incubation for 5–30 min in WT *versus* LRP1 KO MEFs were evaluated by two-way ANOVA. *P < 0.05. Representative images of confocal microscopy analysis for Wnt5a and LRP1 in WT and LRP1 KO MEFs (*n* = 3) (**e**) or human normal chondrocytes (*n* = 3) **f** Cells were incubated with 20 nmol/L Wnt5a for 3 h in the absence (**g**, **h**) or presence of 500 nmol/L RAP (**h**). Wnt5a, LRP1, cytoskeleton and nucleus were visualised as described under “Materials and Methods”. Scale bar, 10 µm. Regions delineated by the white squares in the panels have been magnified in the top right (**g**). WT and LRP1 KO MEFs (*n* = 3) were incubated with 20 nmol/L Wnt5a for 3–24 h and Wnt5a in the medium and cell lysate were detected by Western blotting (**i**). Densitometric analysis of immunoreactive Wnt5a bands was carried out. The relative amount of Wnt5a in the media, cell lysate and both media and cell lysate (total) were expressed by taking the amount of Wn5a after 3-h incubation in WT MEFs as 1 (**j**). **k** human normal chondrocytes (*n* = 3) were incubated with 20 nmol/L Wnt5a for 1–24 h and analysed as in **a** and **b**. The relative amount of Wnt5a after 24-h incubation was expressed by taking the amount of Wn5a after 1-h incubation as 1. Circles represent individual experiment and bars show the mean ± *SD*

The observed skeletal defects could potentially arise from a range of mechanisms including β-catenin-dependent canonical and non-canonical Wnt/PCP signalling pathways. β-catenin deficiency in mice results in fused synovial joints,^45^ delayed ossification^46,47^ and increased osteoclastogenesis.^48,49^ Accumulating evidence suggests a role of LRP1 in the regulation of canonical Wnt signalling.^22,50–52^ Our TOPFlash β-catenin-responsive luciferase reporter assay in WT and LRP1 KO mouse embryonic fibroblasts (MEFs) also showed that LRP1 loss reduced activation of canonical Wnt pathway by exogenously added Wnt3a (Fig. S4). However, little is known about a role of LRP1 in Wnt/PCP pathway. The markedly thicker and shorter long bones (Fig. 4), and disrupted growth plate with disorganised columnar chondrocytes (Figs. 2 and 5) closely resemble phenotypes associated with defects in Wnt/PCP signalling pathway (Fig. 6a).^53^ For example, deletion of core components of Wnt/PCP including Ror2,^54^ Vangl2,^55^ Ryk^56^ and Wnt5a^57^ as well as inducible Wnt5a overexpression^58^ all resulted in shorter and thicker long bones. Critical to interpret these unique bone phenotypes caused by *Lrp1* deficiency in skeletal progenitors but not in chondrocytes, our recent study identified >50 novel candidate binding partners for LRP1 in cartilage^9^ including Wnt5a and Wnt11, Wnt/PCP core components. We therefore investigated the direct interaction of Wnt5a and Wnt11 with LRP1 using a solid-phase binding assay. The assay employed purified Wnts and full-length LRP1.^59^ We found that Wnt5a and Wnt11 directly bind to immobilised LRP1 with high affinity (apparent binding constant (*K*_*D,app*_) of 31 nmol/L and 42 nmol/L, respectively (Fig. 6b, c). In contrast, binding of Wnt3a, a key canonical Wnt component, to LRP1 was negligible with *K*_*D,app*_ of >200 nmol/L (Fig. 6d).

Since Wnt5a dysregulation may explain the malformation of long bones, function of LRP1 in cellular trafficking of Wnt5a was investigated. To test whether LRP1 facilitates cell-association of Wnt5a through direct capture of Wnt5a, exogenously added Wnt5a in the cell-lysate of WT and LRP1 KO MEFs^60^ was monitored for incubation periods ranging between 0-60 min. The levels of cell-bound Wnt5a in WT MEFs after incubation for 5, 15 and 30 min were significantly higher than these in LRP1 KO cells with 1.6, 1.5 and 1.4-fold differences, respectively, whereas no significant difference in the cell-bound Wnt5a levels was observed after 60 min (Fig. 6e, f). These results suggest that Wnt5a binds to the cells without LRP1 but LRP1 facilitates its initial cell-association. We next examined internalisation of Wnt5a and Wnt11 in these cells. Immunofluorescent confocal microscopy analysis revealed the co-localisation of LRP1 and Wnt5a (Fig. 6g) or Wnt11 (Fig. S5A) inside WT MEFs. The fluorescent signal of Wnt5a and Wnt11 was markedly reduced in the LRP1 KO MEFs, indicating that LRP1 is responsible for their cellular uptake. The intracellular colocalisation of exogenously added Wnt5a and LRP1 was also observed in human primary chondrocytes, whose LRP1 levels are higher than WT MEFs (Fig. 6h). Receptor-associated protein (RAP), which antagonises ligand binding by competitively occupying the ligand binding region of LRP1, markedly reduced internalisation of Wnt5a.

Several secreted LRP1 ligands are degraded intracellularly following internalisation.^6–10^ Some ligands, however, are recycled back to the extracellular milieu, facilitating their distribution and availability.^61,62^ Our previous secretome analysis found that Wnt5a is not increased in the chondrocyte culture medium upon inhibition of LRP1-mediated endocytosis,^9^ suggesting a possibility of endocytic recycling of Wnt5a. We thus monitored the level of exogenously added Wnt5a in the medium and cell lysate of WT and LRP1 KO MEFs by Western blot analysis. After 3–24 h incubation, exogenously added Wnt5a was detected in both the conditioned medium and cell lysate (Fig. 6j, k). No significant difference in Wnt5a levels were observed in LRP1 KO compared to WT MEFs and relatively large amounts of Wnt5a still remained even after 24-h incubation with the cells. Similar results were also observed for Wnt11 (Fig. S5B, C). In human chondrocytes, approximately 26% of exogenously added Wnt5a was degraded after 24-h incubation with the cells (Fig. 6l). These results suggest that LRP1 facilitates initial cell-association and mediates internalisation of Wnt5a but its endocytic clearance is likely dependent on the cell-type and not as rapid as other LRP1 ligands such as TIMP3,^5,6^ MMP13^7^ and a disintegrin and metalloproteinase with thrombospondin motifs (ADAMTS)5.^10^

### LRP1 partially colocalises with Wnt5a and its deficiency alters abundance and distribution of Wnt5a in the developing limbs

**Fig. 7:**
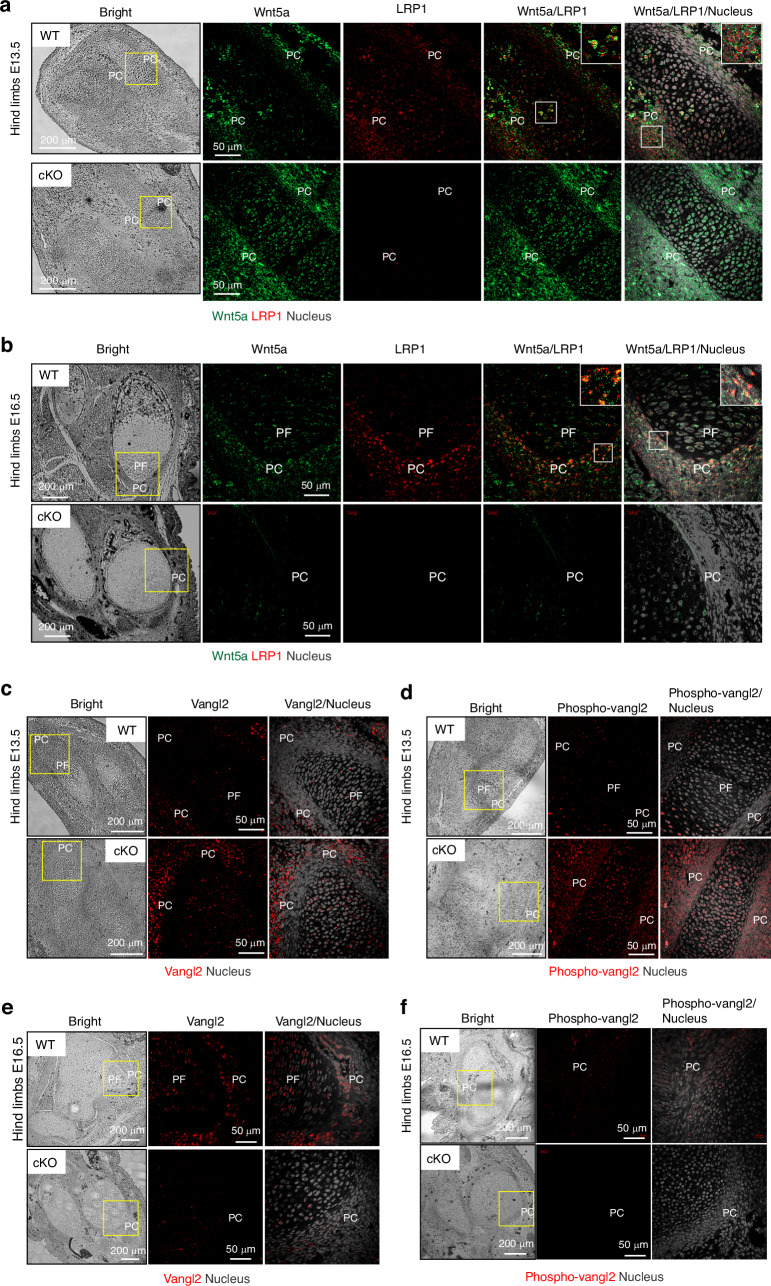
LRP1 partially colocalises with Wnt5a and its deficiency alters abundance and distribution of Wnt5a in the developing limbs. Representative images of confocal microscopy analysis for Wnt5a and LRP1 (**a** and **b**), and total (**c** and **e**) and phosphorylated Vangl2 (**d** and **f**) in E13.5 (**a**, **c** and **d**) or E16.5 (**b**, **e** and **f**) hind limb sections of WT and *Lrp1*^flox/flox^*/Prrx1*^Cre^ (cKO) mice (*n* = 3). Wnt5a, LRP1, Vangl2, phospho-Vangl2 and nucleus were visualised as described under “Materials and Methods”. Regions delineated by the white squares have been magnified in the top right of each panel. PF proliferative flattened chondrocytes; PC perichondrium. Scale bar, 50 µm

We further investigated the interaction of LRP1 and Wnt5a and how LRP1 deficiency affects Wnt5a distribution and activity in the developing limbs. Wnt5a mRNA is expressed in the perichondrium in developing limbs^57,63,64^ but Wnt5a protein distribution in the developing limbs remains incompletely understood. Our immunofluorescent confocal microscopy analysis Wnt5a protein in E13.5 and E16.5 revealed a partial colocalisation of Wnt5a and LRP1 in E16.5 hind limbs (Fig 7a, b). Wnt5a immunosignal was increased in E13.5 *Lrp1*^flox/flox^*/Prrx1*^Cre^ compared to WT limbs (Fig. 7a). In contrast, it was substantially reduced in E16.5 *Lrp1*^flox/flox^*/Prrx1*^Cre^ compared to WT limbs (Fig. 7b). To evaluate Wnt5a activity, we examined expression and phosphorylation of Vangl2, a core component of Wnt/PCP. It has been reported that the abundance of Vangl2 is tightly controlled by the ubiquitin-proteasome system through endoplasmic reticulum–associated degradation.^65^ Wnt5a activity prevents proteasomal degradation of Vangl2, facilitating its export from the endoplasmic reticulum to the plasma membrane. Wnt5a also dose-dependently induces phosphorylation of Vangl2 through Ror2 to establish Wnt/PCP gradient.^55^ Immunofluorescent confocal microscopy analysis revealed substantial increase in total and phosphorylated Vangl2, respectively, in E13.5 *Lrp1*^flox/flox^*/Prrx1*^Cre^ compared to WT limbs (Fig. 7c, d). In contrast, remarkable reduction of Vangl2 with its phosphorylated form almost absent in E16.5 *Lrp1*^flox/flox^*/Prrx1*^Cre^ compared to WT limbs (Fig. 7e, f).

To investigate the regulatory role of LRP1 in Vangl2, we examined the effect of siRNA-mediated gene-knockdown of *LRP1* on Vangl2 mRNA and protein levels. Neither mRNA nor protein levels of Vangl2 was affected by the knockdown of LRP1 in human chondrocytes (Fig. S6A, B), suggesting that LRP1 indirectly affects Vangl2 protein levels potentially via Wnt5a regulation. Confocal microscopy analysis of LRP1 and Vangl2 in human chondrocytes further revealed their little colocalisation (Fig. S6C).

### LRP1 regulates Wnt/PCP signalling in *Xenopus* embryonic development

Finally, we evaluated the role of LRP1 for non-canonical Wnt signalling using *Xenopus laevis* (African clawed frog), which is an invaluable model system for studying the role of Wnt signalling in development.^66^ In *Xenopus*, convergent extension movements are known to be controlled by non-canonical Wnt/PCP signalling.^67,68^ Loss of expression or overexpression of WNT/PCP components thus leads to embryos showing shortened trunks (Fig. S7A).^67,68^ We also confirmed that injection of Wnt5a or Wnt11 mRNAs into the dorsal marginal zone of 4-cell stage embryos results in shortened tadpoles (Fig. S7B, C).

**Fig. 8:**
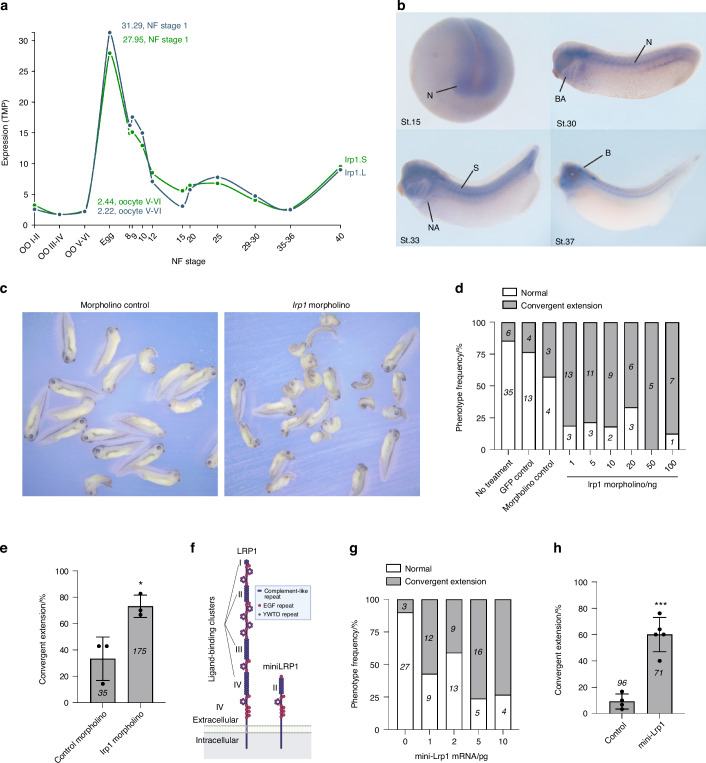
LRP1 regulates Wnt/PCP signalling in *Xenopus* embryonic development. **a** *lrp1* gene expression profile during *Xenopus laevis* embryonic development available in Xenbase. NF, Nieuwkoop and Faber. **b** *lrp1* wholemount in situ hybridisation during the development. N neural tissue, BA branchial arches, S somites, NA neuroadrenergic cells; **b**, brain. Various doses (**d**) or 20 ng (**c** and e) of *lrp1* morpholino were injected into 1 cell of 4-cell stage embryos in the dorsal marginal zone. Representative images of Xenopus tadpoles treated with control and *lrp1* morpholino after fixation at NF stage 35/36 (**c**). Normal embryos and those with convergent extension phenotype were counted and percentages for frequency of each phenotype (**d**) or convergent extension phenotype in three independent experiments (**e**) were shown. Circles represent individual experiment and bars show the mean ± *SD*. *P < 0.05 by 2-tailed Student’s *t* test. Number of embryos counted is stated in each bar graph. **f** Schematic representation of full-length LRP1 and the LRP1 mini-receptor consisting of the ligand-binding cluster II and the entire C-terminus, including the transmembrane domain and the cytoplasmic tail. Illustration created with BioRender (https://biorender.com). Various doses (**g**) or 5 pg (**h**) of mini-*Lrp1* mRNA were injected and the phenotypes were counted as in **d** and **e**. Circles represent individual experiment and bars show the mean ± *SD*. ***P < 0.001 by 2-tailed Student’s *t* test. The number of embryos counted is stated in each bar graph

According to Xenbase, *lrp1* gene expression in Xenopus laevis starts at Oocyte V-VI stage, peaks at Nieuwkoop and Faber stage 1 and then remains on throughout development (Fig. 8a).^69,70^ Wholemount in situ hybridisation using a *lrp1* probe showed that *lrp1* is expressed in the neural tube, branchial arches, somites and neuroadrenergic cells during the development (Fig. 8b). Gene-knockdown of *lrp1* by injection of various doses of lrp1 morpholino into the dorsal side of 4-cell stage embryos caused conversion extension phenotypes of shortened trunks at all the concentrations tested (Fig. 8c, d). Compared to control morpholino, 20 ng of lrp1 morpholino increased frequency of conversion extension phenotype by ~2.2-fold (Fig. 8e**)**. We next examined the effect of overexpression of LRP1 mini-receptor consisting of the ligand-binding cluster II and the entire C-terminus, including the transmembrane domain and the cytoplasmic tail (Fig. 8f). This functional mini-LRP1 receptor maintains capacity to mediate clathrin-dependent endocytosis of LRP1 ligands.^71^ Injection of the mini-LRP1 increased frequency of conversion extension phenotype in a dose-dependent manner (Fig. 8g). Compared to control, 5 pg of mini-*Lrp1* injection increased frequency of conversion extension phenotype by ~8.2-fold (Fig. 8h). These results suggest a role for LRP1 in regulation of the Wnt/PCP pathway.

## Discussion

This study showed, for the first time, a critical role of LRP1 in skeletal progenitor cells and its regulation of the Wnt/PCP pathway. *Lrp1* deletion in early skeletal progenitors caused severe defects in multiple bones and joints which persisted into skeletally mature 14-week-old mice, indicating a non-redundant function in skeletal development and maturity. These observations of long bone and joint malformations were not evident in *Lrp1*^flox/flox^*/Col2a1*^Cre^ mice^27^ or *Lrp1*^flox/flox^/*Acan*^CreERT2^ mice. In our *Lrp1*^flox/flox^*/Prrx1*^Cre^ mice, *Prrx1*^Cre^ gene expression starts at E9.5^35–38^ in early skeletal progenitor cells, whereas in the *Lrp1*^flox/flox^*/Col2a1*^Cre^ strain, *Col2a1*^Cre^ gene expression was confirmed in chondrocytes in limbs at as early as E12.5.^40^ These results suggest that both timing and cell type of LRP1 deficiency contribute to profound differences in their phenotypes. It is likely that LRP1 in early skeletal progenitor cells including perichondrium rather than chondrocytes and prior to E12.5 is more intrinsically involved in guiding proper formation of multiple cartilage and bone elements. Similarly, mice, in which Wnt5a overexpression is induced at E10.5 exhibited a much more severe long bone phenotype than mice induced at E12.5.^58^ Indeed, overexpression of Wnt5a at E13.5 does not result in visible bone phenotype, indicating that the most critical period for Wnt5a in limb development is prior to E13.5. Our study demonstrates the regulation of Wnt/PCP signalling pathway by LRP1 during this critical early time point.

The observed skeletal defects could potentially arise from a range of mechanisms, which require further investigations. Our data demonstrate that TIMP3 and CCN2 are tightly regulated LRP1 ligands,^6,9^ which accumulated throughout the limb in P0 when LRP1 was deleted in *Lrp1*^flox/flox^/*Prrx1*^Cre^ mice. Accumulation of these ligands can affect bone growth^72–74^ but given that many other LRP1 ligands could be accumulated in the tissue, their effect may mitigate the presence of single matricellular protein. Fused synovial joints,^45^ delayed ossification^46,47^ and increased osteoclastogenesis^48,49^ can be explained by a role of LRP1 in the regulation of canonical Wnt signalling. *Lrp1* deletion in mouse neural crest cells results in heart defects, which are associated with a decrease in canonical Wnt signalling.^22^ In vitro, LRP1 interacts with Frizzled1 and downregulates canonical Wnt pathway in HEK293T cells.^50^ In contrast, LRP1 stimulates canonical Wnt pathway and prevents intracellular cholesterol accumulation in fibroblasts.^51^ We also found that LRP1 loss reduced activation of canonical Wnt pathway by exogenously added Wnt3a in MEFs, suggesting a facilitation of canonical Wnt pathway by LRP1. Macrophage LRP1 also increases canonical Wnt pathway by directly binding to and effectively removing secreted frizzled-related protein 5, which prevents Wnt binding to its receptor.^52^ Importantly, Wnt5a can activate or inhibit canonical Wnt signalling pathway depending on their receptor availability.^75^ The role of Wnt5a and LRP1 interaction in regulation of canonical Wnt signalling warrants further investigations but these studies emphasise the developmental stage and tissue-specific nature of Wnt signalling and LRP1 function. Considering that *Lrp1* deficiency in skeletal progenitor cells, osteoblasts^28^ and osteoclasts^29^ results in increased osteoclastogenesis, LRP1 might be selectively regulating canonical Wnt pathway during osteoclastogenesis.

**Fig. 9:**
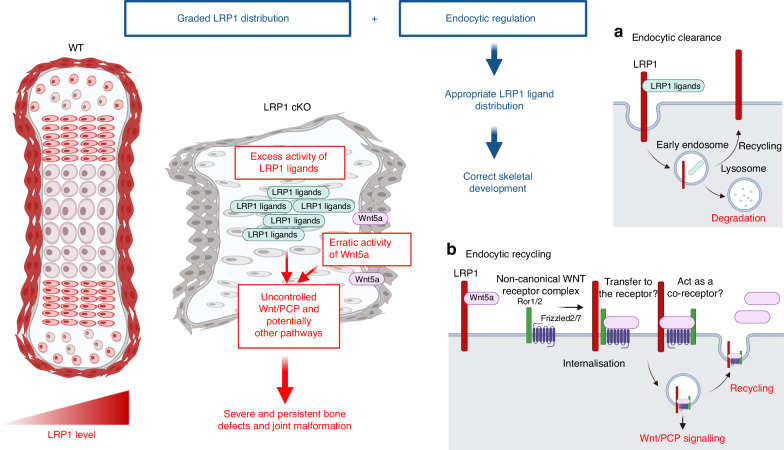
A novel and critical role for LRP1 in skeletal development and its deficiency in emergence of skeletal pathologies. Skeletal progenitor LRP1 is likely to have at least two different functions: to remove molecules from extracellular milieu and degrad them intracellularly (**a**), and to capture, recycle and distribute molecules (**b**). Combination of graded distribution of LRP1 and LRP1-mediated endocytosis regulates distribution of extracellular signalling molecules. This provides a novel mechanism for appropriate distribution of extracellular signalling molecules to ensure that bone and joint form correctly. Thus, loss of LRP1 leads to excess activity of some of LRP1 ligands and erratic Wnt/PCP signalling, resulting in severe defects in multiple bones and joints. Illustration created with BioRender (https://biorender.com)

Skeletal progenitor LRP1 has at least two different functions: to remove molecules from the extracellular milieu and target them for intracellular degradation, and to capture, recycle and distribute molecules (Fig. 9). Heparan sulfate proteoglycans are known to facilitate formation of extracellular gradient of morphogens by capturing them from extracellular milieu.^76^ Otherwise, these morphogens can be freely diffused in tissue. Furthermore, Kawata et al. showed that LRP1 mediates the transcytosis of CCN2 and may determine its gradient distribution in the developing bone.^61^ Considering that LRP1 captures exogenously added Wnt5a, loss of LRP1 may lead to diffusion of Wnt5a in the tissue. We hypothesised that the different effects of LRP1 loss on Wnt5a levels in E13.5 and E16.5 limbs are caused by its endocytic clearance by E13.5 chondroprogenitor cells in bone template and endocytic recycling of Wnt5a by E16.5 perichondrial cells. On the other hand, LRP1 phosphorylation is likely to be dispensable for its function in skeletal development since knock-in mouse models of mutation of the proximal, and separately, distal NPxY motif to disable phosphorylation exhibited normal skeletal development.^77^

This study identifies LRP1 as a major receptor for Wnt5a. Higher levels of cell-associated Wnt5a were detected in WT compared with LRP1 KO cells, suggesting that LRP1 effectively captures Wnt5a from the cellular microenvironment. It has been reported that Wnt5a is internalised by cells via a clathrin-dependent pathway.^78,79^ We found that LRP1 mediates Wnt5a endocytosis but internalised Wnt5a is not rapidly degraded, and is likely recycled. Our immunofluorescence staining further revealed a partial colocalisation of LRP1 and Wnt5a in bone template and perichondrium of E13.5 and E16.5 WT limbs. Strikingly, LRP1 deficiency results in opposite effects on Wnt5a levels in E13.5 and E16.5 limbs with elevation in E13.5 and almost entire absence in E16.5. These results suggest that cell type- and developmental stage-dependent function of LRP1 in regulation of Wnt5a, emphasising the complexity and diversity of skeletal progenitors and importance of further research. This study also showed an association of Wnt5 and Vangl2 levels. Considering no significant effect on LRP1 knockdown on Vangl2 levels and their different cellular localization, LRP1 may be indirectly affecting Vangl2 protein levels via Wnt5a regulation. To understand how LRP1 regulates Wnt/PCP signalling pathway, we are currently exploring a possibility that LRP1 facilitates Wnt5a binding to its cell-surface receptor complexes consisting of Ror1/2 and the frizzled receptors (Fig. 9b).^80–82^

The perichondrium is a dense layer of fibrous connective tissue that covers cartilage in endochondral ossification. Two-way signalling between cells in the perichondrium and the underlying cartilage are essential for endochondral bone formation but questions remain regarding molecular mechanisms underpinning cellular communication between them.^34,53^ Our present study revealed an abundant expression of LRP1 in the perichondrium, strongly suggesting a role for LRP1 in the signalling pathways underpinning bone formation. Furthermore, the colocalisation of LRP1 and Wnt5a in the perichondrium raises the possibility that these two molecules interact to control the recruitment of chondroprogenitor cells from the perichondrium into growth plate. The inability of disorganised columnar chondrocytes and/or possible uncontrolled recruitment of chondroprogenitor cells caused by LRP1 deficiency may contribute to skeletal element widening.

It is worth noting that the data presented here differ significantly from other LRP family members associated with skeletal formation, such as LRP5 and LRP6, which function as Wnt coreceptors with the frizzled receptor and regulate canonical Wnt/β-catenin signalling.^83,84^ LRP5/6 interact with many Wnts (Wnt1/2/3/3a/2b/6/8a/9a/9b/10b) but not Wnt5a or Wnt11.^85^ *Lrp6* KO mice die at birth and exhibit a variety of severe developmental abnormalities resembling those caused by mutations in Wnt1, Wnt3a and Wnt7a.^83^ *Lrp6* KO limbs showed a consistent loss of the most posterior digit but did not exhibit the limb outgrowth defects observed in *Wnt5a* KO mice.^57^ Some *Lrp6* KO mice exhibited deletion of additional digits and the radius, as well as malformation of the ulna. Therefore, *Lrp1*^flox/flox^*/Prrx1*^Cre^ mice *Lrp6* KO phenotypes are distinct.

Since the 2.4 kb *Prrx1* enhancer is reported to be expressed in skeletal muscle as early as E16.5,^37^ we cannot exclude the possibility that skeletal muscle LRP1 plays a role in the observed phenotypes, in particular, unopened digits in hind limb and twisted long bones. Since fusion of digits was not observed in hind limb of *Lrp1*^flox/flox^*/Prrx1*^Cre^ mice, muscle dysfunction might contribute to their closed hind limb fingers. Twisted bones can be explained by altered mechanical adaptation due to abnormal skeletal muscle-bone interaction. *Lrp1*^flox/flox^/*Prrx1*^Cre^ mice exhibited significant differences in limb length and femur thickness as early as at P0. One possibility to rationalise drastic skeletal changes at this stage is the mechanical stimuli generated by the embryo’s active movements. Embryos with restricted movements in utero showed a skeletal phenotype of reduced ossification, especially in forelimbs.^86^

In conclusion, we showed that abundant expression of LRP1 in early skeletal precursor cells, its critical role for synovial joint formation and accurate bone growth. We further demonstrated the regulation of Wnt/PCP signalling pathway by LRP1 which may explain the malformation of long bones in *Lrp1*^flox/flox^/*Prrx1*^Cre^ mice. We propose that combination of graded distribution of LRP1 and LRP1-mediated endocytic clearance and recycling of extracellular signalling molecules provides a novel mechanism for appropriate morphogen gradient formation to ensure that bones and joints form correctly. Investigations into the mechanisms underpinning formation of severe and persistent defects in skeletal elements caused by LRP1 loss are an essential for understanding the fundamental processes of morphogenesis, as well as the emergence of skeletal pathologies including DDH, osteoporosis and OA.

## Materials and Methods

### Mice

All experiments were carried out in accordance with relevant laws and institutional guidelines at the University of Liverpool, with full ethical review and approval, compliant to UK Home Office regulations (PP2097059). The *Lrp1*^flox/flox^*/Prrx1*^Cre^ mice were generated by crossing the *Lrp1*^flox/flox^ (Strain 012604, the Jackson lab) and *Prrx1*^Cre^ (Strain 005584, the Jackson lab) mice. For post-natal analysis, 22 homozygotes (*Lrp1*^flox/flox^*/Prrx1*^Cre^, 11 males and 11 females), 14 heterozygotes (*Lrp1*^flox/wt^*/Prrx1*^Cre^, 11 males and 13 females) and 21 WT littermates (*Lrp1*^flox/flox^ or *Lrp1*^wt/wt^*/Prrx1*^Cre^, 10 males and 11 females) were examined for this study. For embryos, we examined 26 homozygotes, 12 heterozygotes and 24 WT littermates were examined for this study.

### Mouse genotyping

For genotyping, mice DNA was extracted from ear notches or tails using the RED ExtractN-Amp Tissue PCR Kit (SIGMA). For *Lrp1*^flox^, following primers were used; forward 5’- CATACCCTCTTCAAACCCCTTCCTG - 3’, reverse 5’-GCAAGCTCTCCTGCTCAGACCTGGA - 3’ with the following PCR conditions: 1 cycle of 94 °C for 3 min, 35 cycles of 94 °C for 30 s; 65 °C for 30 s; 72 °C for 30 s, ended with a final cycle of 72 °C for 2 mins. For *Prrx1*^Cre^, following primers were used, forward 5’- GCTGCCACGACCAAGTGACAGCAA - 3’, reverse 5’- CAGTAGCCTCATCATCACTAGATG - 3’ with the following PCR conditions: 1 cycle of 94 °C for 1 min, 40 cycles of 94 °C for 30 s; 60°C for 30 s; 72 °C for 1:30 mins; followed by a final cycle of 72 °C for 5 mins.

### Immunohistochemistry

Slide sections were dewaxed and rehydrated by xylene and decreasing ethanol concentrations. Epitope unmasking was performed using a basic antigen retrieval reagent (R&D systems). The slides were immersed in the basic reagents and kept in a water bath for 10 minutes at 95 °C. To block the endogenous peroxidase activity, 0.3% hydrogen peroxide was added to the slides and the slides were kept at 37 °C for 15 minutes. Avidin/Biotin blocking kit (Vector Lab) was used to block endogenous biotin, biotin receptors and avidin. To block unspecific antibody binding, 10% goat serum was added to the slides and incubated for 3 h at room temperature (RT). The primary antibody was then added to the slides and kept at 4 °C overnight. The impress HRP Goat anti-Rabbit IgG polymer Detection Kit (Vector Lab) was used as a secondary antibody. One drop was used for each section, and the slides were kept at RT for 30 minutes. For signal enhancement, the VECTASTAIN ABC-HRP Kit (Vector Lab) was added to the slides and kept in the dark for 30 minutes. For signal visualisation, the DAB substrate kit (Vector Lab) was used to develop the brown signal. Fast green was used for counterstaining. Slides were dehydrated, cleared and mounted using increasing concentrations of ethanol, xylene and DPX (SIGMA), respectively. The primary antibodies used were as follows; rabbit monoclonal anti-LRP1 (1:200)(ab92544, Abcam), rabbit polyclonal anti-Sox9 (1:500)(AB5535, SIGMA), rabbit polyclonal anti-Wnt5a (1:200)(bs1948R, Bioss). The Rabbit IgG control antibody (I1000, Vector Lab) was used as isotype control. At least three mice/group were analysed for each staining.

### Microscopic examination of tissue sections

For microscopic examination and imaging, the Nikon Eclipse Ci microscope with the DS-Fi2 high-definition colour camera head was used. All images were visualised using NIS-Elements imaging. The Zeiss Axio Scan.Z1 slide scanner system was used for the automated imaging. The resulting images were inspected using ZEN 3.0 (Blue edition), in which the image scale bar was added.

### Histology and staining

All samples were fixed with neutral buffered formalin overnight, then kept in 70% ethanol until processing. EDTA was used for bone decalcification with different incubation times based on the samples age. Sample processing was automated using the Leica ASP300 tissue processor (Leica Microsystems, UK). The Leica EG1150 H embedding station was used for sample embedding. Sample sectioning at 5 µm thickness was performed using the Leica RM2245 microtome. For H&E staining, slides were deparaffinised in xylene and rehydrated was performed using decreasing ethanol concentrations. For nucleus staining and counterstaining, slides were stained with haematoxylin and eosin, respectively, for 5 minutes. The slides were then dehydrated in increasing ethanol concentrations before clearing them in xylene followed by mounting. For Safranin O staining, sections were deparaffinised, rehydrated and stained with haematoxylin for 30 seconds followed by counterstaining with 2% fast green for 2 minutes. The sections were then dipped in acetic acid for 20 seconds before staining with safranin O for 8 minutes. At least three mice/group were analysed for each staining.

### HCR

E10.5 WT and *Lrp1*^flox/flox^*/Prrx1*^Cre^ mouse embryos were fixed with 4% RNase free paraformaldehyde (PFA) in 4 °C overnight. HCR v3.0 was performed as described by Choi et al.^39^ with slight modifications using molecular instruments. Briefly, fixed embryos were immediately dehydrated with sequential addition of increasing concentrations (25%, 50% and 75%) of methanol in PBS until reaching 100% methanol for storing at −20 ^o^C. When required, embryos were rehydrated with decreasing concentrations (75%, 50% and 25%) of methanol in PBS plus 0.1% Tween (PBST). Embryos were then permeabilised in 10 µg/mL proteinase K (ThermoFisher) in PBST for 20 minutes. Embryos were post-fixed with 4% PFA in PBS for 20 min at RT, followed by two washes with PBST for 5 minutes each on ice. After one wash with a solution consisting of 50% of 5x saline sodium citrate with 0.1% tween 20 (SSCT) and 50% of PBST for 5 minutes and a second wash with SSCT for 5 minutes, embryos were incubated with probe hybridisation buffer consisting of 30% formamide, SSCT, 9 mmol/L citric acid (pH 6.0), 50 µg/mL heparin, Denhardt’s solution and 10% dextran sulfate on ice for 5 min, followed by incubation at 37 °C for 30 minutes. Then, embryos were incubated overnight in HB1 with DAPI and each 20 of RNA split initiator probe pairs for *Lrp1* and *Fgf8* (Sigma) at 37 °C for overnight. Details for probes and amplifiers were provided in Supplementary table. Embryos were then washed five times with 30% probe wash buffer consisting of 30% formamide, SSCT and 50 µg/mL heparin for 15 minutes each. B3 hairpin 1 and hairpin 2 conjugated to an Alexa Fluor 594 and B5 hairpin 1 and hairpin 2 conjugated to an Alexa Fluor 488 were snap cooled by heating to 95 °C for 90 seconds and cooled in the dark for 30 minutes before being mixed and incubated with amplifier buffer consisting of SSCT and 10% dextran sulfate at 1:50 ratio for overnight. Embryos were washed extensively with SSCT and were imaged on an Andor Dragonfly spinning disc confocal microscope mounted on an inverted Leica DMi8 base using a 10 × 0.45 NA air objective. DAPI and Alexa Fluor 488 and 594 were sequentially excited with 405, 488, and 561 nm laser diodes respectively, and emitted light reflected through 450/50 nm, 525/50 nm and 600/50 nm bandpass filters, respectively. An iXon 888 EM-CCD camera was used to collect emitted light, and data were captured using Fusion version 5.5 (Bitplane) followed by analysis using Imaris viewer 10.1.0.

### μCT

For ex vivo high-resolution μCT imaging, all samples were fixed in buffered and then kept in 70% ethanol until processing. µCT scanning was performed using the Skyscan 1272 (SKYSCAN) for all developmental stages. Embryos and postnatal samples were scanned at a resolution of 4.5 µm (60 kV, 150 µA, rotation step size 0.3°, 0.5 mm aluminum filter and 4-frame averaging). Skyscan NRecon software was used to reconstruct the obtained images. Skyscan Data Viewer software was used to measure bone length and width. For trabecular bone, the Skyscan CT-analyser software was used to identify the trabecular bone in the tibia. CTvox software was used to visualise the obtained images in a 3D form.

For in vivo μCT, the University of Liverpool’s centre of preclinical imaging provided the imaging services using the Quantum GX-2 system (PerkinElmer). Whole-body scan images were acquired with the protocol FOV72, high speed, 8 sec x 3. High-resolution scans for upper and lower limbs were performed with the protocol FOV36, high resolution, 4 min. At least five mice/group were analysed for each staining.

### Locomotor activity monitoring

All mice were housed in a Digital Ventilated Cage (DVC®) rack, equipped with a home cage monitoring system capable of automatically measuring animal activity 24/7.^87^ The DVC® rack is installed on a standard IVC rack (Tecniplast DGM500) by adding sensing technologies externally to the cage, so that neither modifications nor intrusion occur in the home cage environment. Animal locomotion activity is monitored via a capacitance sensing technology by means of 12 contactless electrodes, uniformly distributed underneath the cage floor. The 6-week-old WT and cKO mice were monitored for 4 weeks and total distance and average speed of each mouse were measured.

### Whole-mount skeletal staining

The whole-mount skeletal staining was done following the Rigueur and Lyons’s protocol.^88^ Briefly, after dissection, adult mice skeletons were initially kept in 95% ethanol for 4 h before changing the solution and leaving in 95% ethanol overnight at RT. Then, skeletons mice were kept in acetone for two days at RT. Cartilage staining was performed by immersion in alcian blue for three days; then, mice underwent two changes of 95% ethanol for 4 h and overnight for destaining. For pre-clearing, skeletons were kept in 1% KOH overnight at 4°C. Bone staining was performed by submersion in alizarin red for five days. Lastly, the final clearing was performed using 1% KOH prior to long-term storage in 100% glycerol.

### Calcein double labelling

Calcein double labelling was performed as described previously.^89^ Two intraperitoneal (IP) calcein injections (150 µL/mouse) were given to 5–6 weeks old mice 4 days before culling at an interval of two days. Mice were dissected, and the tibia bones were fixed in natural buffered formalin and kept in 70% ethanol until processing. The bones were dehydrated at 4 °C with xylene and decreasing ethanol concentrations. Sample infiltration was performed under vacuum for seven days using a solution containing 88.99% Methyl Methacrylate (MMA), 10% dibutyl phthalate, 1% Perkadox 16 and 0.01% Novoscave. Teflon blocks filled with MMA were used for bone embedding. The blocks were kept at 30 °C in a water bath to polymerise for 18 h. After that, Historesin was used to attach the embedding rings. Sectioning at 5 µm was performed using the Leica RM2265 microtome. To analyse bone formation, sections were stained without de-plasticisation with 0.1% calcein blue for 3 minutes, dehydrated using different ethanol concentrations and cleared with xylene changes. Three mice/group were analysed using the CalceinHisto software.^89^

### Tartrate-resistant acid phosphatase (TRAP) staining

Samples were embedded and sectioned as described for calcein double labelling analysis. Bone section de-plasticisation was performed by three changes of 2-methoxyethyl acetate (MEA). Section clearing and rehydration were accomplished by xylene and decreasing concentrations of ethanol. TRAP solution was prepared by dissolving naphthol ASTR-phosphate (1.4 mg/mL) and fast red (1.4 mg/mL) in a 0.2 mol/L acetate buffer (pH 5.2) containing 100 mmol/L sodium tartrate. Bone sections were kept in the TRAP solution for 2 h at 37 °C. Then bone sections were counterstained with 0.33 g/L aniline blue and 6 g/L phosphotungstic acid for 15 minutes. The slides were washed with distilled water and then cover slipped with Apathy’s serum. Three mice/group were analysed using the TrapHisto program.^89^

### Enzyme-linked immunosorbent assay (ELISA)

Purified human full-length LRP1 (10 nmol/L in 100 µL of 50 mmol/L Tris-HCl (pH 7.5)/150 mmol/L NaCl/10 mmol/L CaCl_2_, TNC) was coated overnight at 4 °C onto microtiter plates (Corning). Wells were blocked with 5% bovine serum albumin in TNC for 24 h at 4 °C and washed in TNC containing 0.1% Brij-35 after this and each subsequent step. Wells were then incubated with various concentrations of Wnts in blocking solution for 30 min at RT. Bound proteins were detected using anti-Wnt3a antibody (ab219412, Abcam), anti-Wnt5a antibody (MAB6452, R&D systems) or anti-Wnt11 antibody (ab31962, Abcam) for 1 h at RT and then with a secondary antibody coupled to horseradish peroxidase for 1 h at RT. Hydrolysis of tetramethylbenzidine substrate (KPL) was measured at 450 nm using a FLUOstar Omega (BMG Labtech). Mean values of technical duplicate were normalized by subtracting the amount of recombinant protein bound to control well that was not coated with LRP1. Extrapolated *K*_*D,app*_ values were estimated based on one-phase decay nonlinear fit analysis using GraphPad Prism 9.

### Monitoring exogenously added Wnt5a and Wnt11 levels in the cell culture

WT and LRP1 KO MEFs were generated as described previously^60^ and kindly provided by Professor Dudley Strickland (University of Maryland School of Medicine). Human normal chondrocytes were prepared as described previously.^9^ For 0–24 h incubation assay, WT and LRP1 KO MEFs, and human chondrocytes (5 × 10^3^/well) were grown in 24 well plate (pre-coated with 0.1% gelatin for overnight) until cells reach confluent. Cells were then incubated with DMEM/F12 for overnight. The medium was replaced with 0.5 mL of fresh DMEM/F12 containing polymyxin B (50 µg/mL), CT1746 (100 μmol/L) and the protease inhibitor cocktail (1/1 000) with or without 20 nmol/L Wnt5a or Wnt11 in the presence or absence of 500 nmol/L RAP. After 0–24 h, 0.5 mL of medium were collected, and the protein was precipitated with trichloroacetic acid and dissolved in 40 μL of 1x SDS-sample buffer (50 mmol/L Tris-HCl pH 6.8, 10 mmol/L dithiothreitol, 2% SDS and 10% glycerol). Cells were washed with phosphate-buffered saline (PBS) once and lysed with 100 μL (for MEFs) or 50 μL (for chondrocytes) of 2x SDS-sample buffer. 12.5 µL of cell lysate and 5 µL (for MEFs) or 10 µL (for chondrocytes) of medium samples were analysed by SDS-PAGE under reducing conditions and underwent Western blotting using anti-Wnt5a antibody (MAB6452, R&D systems) or anti-Wnt11 antibody (ab31962, Abcam). Immune signals for exogenously added Wnt5a or Wnt11 in the medium and cell lysate were quantified using ImageJ. Average actin signal was taken as 1 and Wnt5a or Wnt11 signal in the cell lysate was normalised against each actin signal. The amount of Wnt5a or Wnt11 after 24-h incubation was expressed as a % of the amount of Wnt5a or Wnt11 after 3 h (for MEFs) or 1 h (for chondrocyte) incubation. For 0–60 minutes incubation assay, WT and LRP1 KO MEFs (5 × 10^3^/well) were grown in 96 well plate (pre-coated with 0.1% gelatin for overnight) until cells reach confluent. Cells were then incubated with DMEM/F12 for overnight. The medium was replaced with 50 µL of fresh DMEM/F12 containing polymyxin B (50 µg/mL), CT1746 (100 μmol/L) and the protease inhibitor cocktail (1/1 000) with or without 20 nmol/L Wnt5a. After 0-60 minutes h, medium was removed, cells were washed with PBS once and lysed with 30 μL of 2x SDS-sample buffer. 10 µL of cell lysate samples were analysed by SDS-PAGE as described as above. Wnt5a immune signals in the cell lysate were quantified and normalised as described above and the amount of Wnt5a before incubation was taken as 100%.

### Immunocytochemical localisation of Wnt5a and LRP1

Cells were grown with DMEM/F12 containing 10% FBS on 8-well Lab-Tek chamber slides (Nunc Lab-Tek Chamber Slide System, Thermo Scientific) precoated with 0.1% gelatin in PBS overnight. Once reaching confluency, the cells were rested in serum free DMEM/F12 for 24 h. Cells were then incubated in DMEM/F12 containing polymyxin B (50 µg/mL), the broad-spectrum hydroxamate metalloproteinases inhibitor CT1746 (100 μmol/L) and the protease inhibitor cocktail (a mixture of aprotinin, bestatin, E-64, leupeptin and pepstatin A, 1/1 000)(P1860, SIGMA) with or without 20 nmol/L Wnt5a in the presence or absence of 500 nmol/L RAP for 3 h at 37 °C. Cells were washed with DMEM/F12 three times and fixed with 4% PFA in PBS for 10 min at RT. PFA was removed and cells were incubated with 100 mmol/L Glycine for 3 min at RT. Cells were washed with PBS twice and incubated with 0.1% Sudan black in 70% ethanol for 2 minutes. Cells were washed with 70% ethanol twice and PBS once, and permeabilised with in TNC containing 0.1% Triton X-100 for 3 minutes at RT. Cells were incubated with 10% goat serum for 1 h at RT followed by three times washing with PBS. Each sample was then incubated with anti-Wnt5a antibody (AF645, R&D systems) and anti-LRP1 antibody (ab92544, Abcam) for overnight at 4 °C. Cells were washed with PBS three times and further incubated with Alexa Fluor 488-conjugated anti-mouse IgG and Alexa Fluor 594-conjugated anti-rabbit IgG (Molecular Probes) for 1 h at RT. Actin was stained with Actin-stain 670 phalloidin (Cell Signalling). Cells were washed with PBS five times and mounted with VECTASHEILD antifade mounting media containing DAPI (2BScientific). Immunofluorescence images were acquired using a Zeiss LSM 800 confocal microscope (Zeiss). Images were processed using Zen 2.6 (blue edition, Zeiss).

### Immunofluorescence tissue staining of LRP1, Wnt5a, Vangl2 and phospho-Vangl2

Dewaxing and antigen retrieval was performed as described above. The slides were incubated with 0.1% Sudan black in 70% ethanol for 2 minutes. The slides were then washed with 70% ethanol twice and once with PBS for Wnt5a, LRP1 and Vangl2, and Tris-buffered saline (TBS) for phospho-Vangl2. To permeabilise the tissue, the slides were incubated with 0.4% Triton X-100 in PBS for Wnt5a, LRP1 and Vangl2, and in TBS for phospho-Vangl2 for 10 minutes at RT. To block unspecific antibody binding, 10% goat serum was added to all slides and incubated for 1 h at RT followed by additional 1 h mouse blocking (MKB 2213, Vector labs) for Wnt5a and LRP1 detection. The slides were washed twice with PBS for Wnt5a, LRP1 and Vangl2, and with TBS for phospho-Vavgl2. Each slide was then incubated with anti-Wnt5a antibody (Sc-365370, Santa Cruz), anti-LRP1 antibody (ab92544, Abcam), anti-Vangl2 antibody (21492-1-AP Proteintech) and anti-phospho-Vangl2-S79/S82/S84 antibody (SAB5701946, Merck) for overnight at 4 °C. The slides were washed four times with PBS for Wnt5a, LRP1 and Vangl2, and with TBS for phospho-Vavgl2. The slides were further incubated with Alexa Fluor 488-conjugated anti-mouse IgG and Alexa Fluor 594-conjugated anti-rabbit IgG (Molecular Probes, Eugene, OR) for 1 h at RT. The slides were washed five times with PBS for Wnt5a, LRP1 and Vangl2, and with TBS for phospho-Vavgl2. The Slides were mounted and immunofluorescence images were acquired and processed as described above. Three mice/group were analysed.

### Xenopus laevis embryonic development model

All experiments were carried out in accordance with relevant laws and institutional guidelines at the University of East Anglia, with full ethical review and approval, compliant to UK Home Office regulations (PP5789738). Embryos were generated as described in.^90^ *Xenopus laevis* embryos were primed by injecting females with 100 U of PMSG into lymph sack 3 to 5 days before requiring the embryos. After 72 h, primed females were injected with 500 U of hCG into lymph sack to induce ovulation. Eggs were collected manually and fertilized in vitro. Embryos were de-jelled in 2% cysteine (w/v) solution until they pack closely together incubated at 18 °C and microinjected in 3% Ficoll into 1 cell at the 2–4 cell stage into the dorsal marginal zone. Embryos were further incubated at 13 ° to 23 °C until the required developmental stage was reached with observation and regular changing of 0.1x MMR. Embryo staging is according to Nieuwkoop and Faber (NF) normal table of Xenopus development.^91^ Wholemount in situ hybridisation was carried out as previously described.^92^ Sense and antisense probes were synthesised for lrp1. GFP, Wnt5a, Wnt11 and mini-LRP1 capped RNA for injections was prepared using T7 and SP6 mMessage mMachine kit (AM1344, ThermoFisher). Translation blocking morpholino antisense oligonucleotides targeting lrp1 (5’-TGCCTTGATCCAGTTCTTGG-3’) was purchased from Gene Tools, LLC in USA. A standard control morpholino (5’-CCTCTTACCTCAGTTACAATTTATA-3’) obtained from GeneTools was used for control injections.

### Statistical analysis

The GraphPad software programme was used for statistical analysis. Significance was calculated using the unpaired Student’s t-test to compare each group and displayed on the statistical charts as *P< 0.05, **P < 0.001, ***P < 0.001 and ****P < 0.000 1. A two-way ANOVA was used for the comparison of cell-associated Wnt5a levels between WT and LRP1 KO MEFs after 5–30 min incubation. The data were represented as mean ± SD.

## Supplementary information

Supplementary information

Movie S1

Movie S2
