# Supplementary material for: Skeletal progenitor LRP1 deficiency causes severe and persistent skeletal defects with Wnt pathway dysregulation
Source: Bone Res. 2025 Jan 26;13:17. doi: 10.1038/s41413-024-00393-x (PMC11770177; doi:10.1038/s41413-024-00393-x)
Supplement: Supplementary file 1 — Supplementary information [file 41413_2024_393_MOESM1_ESM.docx]

**Supplementary Information**

**This PDF file includes:**

Supporting methods

Table 1

Figures S1 to S7

Movies S1 and S2

**Supplementary methods**

*Establishment of cartilage Lrp1 conditional KO mice (Lrp1*^flox/flox^*/Acan*^CreERT2^*)*

The Lrp1/Acan mice were developed by mating the Acantm1(Cre-ERT2)Crm/J (strain 019148, the Jackson laboratory) and Lrp1flox (Strain 012604, the Jackson laboratory) mice. These *Lrp1*/*Acan* mice were tamoxifen-inducible. Therefore, upon tamoxifen's timely administration, the *Lrp1* gene was deleted in cartilaginous tissues in the generated offspring. Total 18 homozygotes and 28 wild-type littermates were examined for this study. These mice were genotyped the same way as the *Lrp1*/*Prrx1* mice. In these mice, the deletion of *Exon 2* in the *Lrp1* gene was also investigated. The following primers were used: forward 5'- TCTTCTACCCTATGAACCATTCCC - 3', and reverse 5'-TCTTGGCTCCTCCAGTGTTC - 3' with the following PCR conditions: 1 cycle of 94°C for 4 min, 40 cycles of 94°C for 30 s; 61°C for 30 s; 72°C for 1.5 min, followed by a final cycle of 72°C for 5 mins.

*Tamoxifen oral gavage*

Fifty mg of tamoxifen-free base (T5648, SIGMA) was suspended in 0.5 ml of 100% ethanol and fully dissolved in 4.5 ml of corn oil (SIGMA) by sonication (final 10 mg/ml)l. Similarly, the progesterone (P3972, SIGMA) was prepared following the same steps. The pregnant female was given either 100 or 200 µl of tamoxifen (1 or 2 mg/mice) and 50 µl progesterone (0.5 mg/mice) orally, gavage, twice in a timely manner to generate the experimental groups. Weight measurements were taken before the gavage administrations and mice termination.

*TOPFlash β-catenin-responsive luciferase reporter assay*

WT and LRP1 KO MEFs were grown in 1 ml of DMEM/F12 containing 10% FBS on 24 well plate (pre-coated with 0.1% gelatin for overnight) until cells reached 50% confluent. Cells were then transiently transfected with either negative control reporter plasmid (Bioscience) or the super 8xTOPFlash reporter (referred thereafter as TopFlash), which contains eight TCF/LEF binding motifs upstream of the *firefly* luciferase gene. The transfection also included the pRL CMV plasmid (Promega), which contains the *renilla* luciferase reporter gene to serve as an internal control. Transfections were performed using lipofectamine 3000 reagent as follows: three transfection mixes were prepared for each well: 1) lipofectamine mix: 0.75 µl of Lipofectamine 3000 was dispensed in 50 µl of Opti-MEM reduced serum medium; 2) negative control mix: 1 µg of negative control reporter, 20 ng of pRL, and 1 µl of P300 were dispensed in 25 µl of opti mem reduced serum medium; and 3) TopFlash mix: 1 µg of TopFlash, 20 ng of pRL, and 1 µl of P300 were dispensed in 25 µl of opti mem reduced serum medium. Each mixture was incubated sepatately for 5 minutes at room temperature. After the incubation, the Lipofectamine mix was combined with either the negative control mix or the TopFlash mix, and the resulting mixtures were incubated for an additional 20 minutes at room temperature to form the transfection complexes. After this incubation period, 50ul of transfection mix was added to its corresponding well and incubated for 6 h. Following the 6 h, the medium was replaced with fresh DMEM/F12 containing 10% FBS for overnight. The next day, the medium was replaced with 0.5 ml of serum free DMEM/F12 for serum starvation. After serum starvation, the cells were treated with 100 ng/ml of Wnt3, Wnt5a and Wnt11 for 24 h. To assess luciferase activity, cells were first washed with PBS and then lysed with 30ul of 1x passive lysis Buffer (Promega). The cell lysates were shaken on an orbital shaker at 100rpm for 20 minutes at room temperature. Luciferase activity from both firefly and rennila were measured by the Dual Luciferase Reporter Assay System kit (E1910, Promega). To obtain luminescence values for each well, 10 µl of the cell lysate were transferred to a white 96 well plate, and 40 µl of the detection mixture (comprising of LARII and Stop & Glo reagents) was added automatically using Glomax Multi luminometer (Promega). Luminescence values were then measured using the same luminometer.

*Monitoring exogenously added Wnt5a and Wnt11 levels in the cell culture*

WT and LRP1 KO MEFs were grown with 1 ml of DMEM/F12 containing 10% FBS on 24 well plate (pre-coated with 0.1% gelatin for overnight) until cells reach confluent. Cells were then incubated with DMEM/F12 for overnight. The medium was replaced with 0.5 ml of fresh DMEM/F12 containing polymyxin B (50 µg/ml), CT1746 (100 µM) and the protease inhibitor cocktail (1/1000) with or without 20 nM Wnt11. After 0-24 hours, 0.5 ml of medium were collected, and the protein was precipitated with trichloroacetic acid and dissolved in 40 μl of 1x SDS-sample buffer (50 mM Tris-HCl pH 6.8, 10 mM dithiothreitol, 2% SDS and 10% glycerol). Cells were washed with PBS once and lysed with 100 μl of 2x SDS-sample buffer. 12.5 µl of cell lysate and 5 µl of medium samples were analysed by SDS-PAGE under reducing conditions and underwent Western blotting using anti-Wnt11 antibody (ab31962, Abcam).

*Immunocytochemical localisation of Wnt11, LRP1 and Vangl2*

Cells were grown with DMEM/F12 containing 10% FBS on 8-well Lab-Tek chamber slides (Nunc Lab-Tek Chamber Slide System, Thermo Scientific) precoated without (for LRP1 and Vangl2) or with 0.1% gelatin (for Wnt11) in PBS overnight. Once reaching confluency, the cells were rested in serum free DMEM/F12 for 24 h. For Wnt11, cells were incubated in DMEM/F12 containing polymyxin B (50 µg/ml), CT1746 (100 µM) and the protease inhibitor cocktail (1/1000)(SIGMA, P1860) with or without 20 nM Wnt11 for 3 h at 37 °C. Cells were washed with DMEM/F12 three times and fixed with 4% paraformaldehyde (PFA) in PBS for 10 min at RT. PFA was removed and cells were incubated with 100 mM Glycine for 3 min at RT. Cells were washed with PBS twice and incubated with 0.1% Sudan black in 70% ethanol for 2 minutes. Cells were further washed with 70% ethanol twice and PBS once, and permeabilised with in TNC containing 0.1% Triton X-100 for 3 minutes at RT. Cells were then incubated with 10% goat serum for 1 hour at RT followed by three times washing with PBS. Each sample was then incubated with anti-Wnt11 (ab31962, Abcam) or anti-Vangl2 (sc-515187, Santacruz) and anti-LRP1 (ab92544, Abcam) for overnight at 4 °C. Cells were washed with PBS three times and further incubated with Alexa Fluor 568-conjugated anti-rabbit IgG (Molecular Probes) for one hour at RT. Actin was stained with Actin-stain 670 phalloidin (Cell Signalling). Cells were washed with PBS five times and mounted with VECTASHEILD antifade mounting media containing DAPI (2BScientific). Immunofluorescence images were acquired using a Zeiss LSM 800 confocal microscope (Zeiss). Images were processed using Zen 2.6 (blue edition, Zeiss).

*siRNA-mediated gene silencing of LRP1 in human chondrocytes.*

siRNA oligonucleotides for *Lrp1* (On-TargetPlus SMARTpool siRNA) and non-targeting oligonucleotides were purchased from Thermo Scientific Dharmacon. Human articular chondrocytes were plated at a density of 5 x 10⁴ cells per well in 24-well plates in DMEM containing 10% FCS and incubated until 50% confluent. Lipofectamine 3000 reagent (Thermo Fisher) was used to transfect cells with siRNA at a final concentration of 20 nM in Opti-MEM. At 6 h after transfection, the Opti-MEM was removed and replaced with serum-free DMEM, and cells were further incubated for 24 h.

**Table 1: Version 3 HCR split-initiator probes for *Lrp1* and *Fgf8*.** The sequences for each split initiator probe, the target gene, and the HCR amplifies are shown. Gene sequences were obtained from the National Center for Biotechnology Information (NCBI) database and are listed 5’ to 3’. Probe numbers indicate their consecutive positions along the target mRNA. B3: B3-Alexa-594; B5: B5-Alexa-488. Lowercase letters indicate the initiator sequence recognised by the HCR amplifier.

| **Gene** | **Amplifier** | **ID** | **Sequence** |
| --- | --- | --- | --- |
| Lrp1 | B5 | LRP1_1a | ctcactcccaatctctataaCTGGCCTGGAGCTCGCACCGCTGCT |
|  |  | LRP1_1b | CTCCCCCGTTTCCGCCTCCTCAGTGaactaccctacaaatccaat |
|  |  | LRP1_2a | ctcactcccaatctctataaTTGGTAGGGGCCTGATGGAGCACTA |
|  |  | LRP1_2b | GGGGGGCGTGGACCCGATGGGGGTGaactaccctacaaatccaat |
|  |  | LRP1_3a | ctcactcccaatctctataaCACAATTGGGAGGAGGCGGGGTGGG |
|  |  | LRP1_3b | TCGGAGCCGCCTCTGGCTGCAAAAAaactaccctacaaatccaat |
|  |  | LRP1_4a | ctcactcccaatctctataaCCCCCTCCCAGGGCGAAGCTCACAG |
|  |  | LRP1_4b | CACCCCTGCTTTACTCCTCGCTCCTaactaccctacaaatccaat |
|  |  | LRP1_5a | ctcactcccaatctctataaGGGTGCGCCCCCTGCCCCCAAATTC |
|  |  | LRP1_5b | AGCCCCCTGGGAAGGGCCTGCTGACaactaccctacaaatccaat |
|  |  | LRP1_6a | ctcactcccaatctctataaCCAGGGGCATAGGTGAAATGGTACA |
|  |  | LRP1_6b | TCTTATCCTTTCCTTAAGCAAAGCGaactaccctacaaatccaat |
|  |  | LRP1_7a | ctcactcccaatctctataaCCTTTATCTTCCTCTCCCCGACTCT |
|  |  | LRP1_7b | CCTCGCCCCCCCCAATTGGGGGGTCaactaccctacaaatccaat |
|  |  | LRP1_8a | ctcactcccaatctctataaGCCCCCACCCTCTGGTCCTGTTACT |
|  |  | LRP1_8b | GCATGGTGTGGGCCGATGCAAACAGaactaccctacaaatccaat |
|  |  | LRP1_9a | ctcactcccaatctctataaCAGCGGCAGCAGCAGCAGCAACGGC |
|  |  | LRP1_9b | AGTGGCCCCGGAGACCAGAGCTGAAaactaccctacaaatccaat |
|  |  | LRP1_10a | ctcactcccaatctctataaAACTGCTTAGGGCTGCAAGTTTTAG |
|  |  | LRP1_10b | ATACAGGTGATTTGGTCTCTGCAGGaactaccctacaaatccaat |
|  |  | LRP1_11a | ctcactcccaatctctataaTCTTTCACCGTCACACCGCCAGCCC |
|  |  | LRP1_11b | GGCTTCATCAGAGCCGTCGGGGCAAaactaccctacaaatccaat |
|  |  | LRP1_12a | ctcactcccaatctctataaTGCACGTCTTGCCATCTGCCTGCAG |
|  |  | LRP1_12b | CATACACGGAACACTCGTCAAAATCaactaccctacaaatccaat |
|  |  | LRP1_13a | ctcactcccaatctctataaCACGTGGTGCAGGCTGAGGGAGATG |
|  |  | LRP1_13b | CGTCAGCCAGTCGATTGCCATCTGCaactaccctacaaatccaat |
|  |  | LRP1_14a | ctcactcccaatctctataaAGACCTGCACCTGCAGGTCCTTGCC |
|  |  | LRP1_14b | CTTCCCATCACTTCCCAGGCTGAAGaactaccctacaaatccaat |
|  |  | LRP1_15a | ctcactcccaatctctataaTTCCGCAGCACCACGGACCCAGAGC |
|  |  | LRP1_15b | TTCATGTGCATAACCAACGTGGTACaactaccctacaaatccaat |
|  |  | LRP1_16a | ctcactcccaatctctataaACACAGATCCTGGCAGCCTCCATTG |
|  |  | LRP1_16b | GTTGACGTGGCCTTGGTGGGTGAGCaactaccctacaaatccaat |
|  |  | LRP1_17a | ctcactcccaatctctataaCCTCATCGCTGTGGTCGTGACAGTC |
|  |  | LRP1_17b | GGCTGGTGCAGTGTGGGTTCTTGGGaactaccctacaaatccaat |
|  |  | LRP1_18a | ctcactcccaatctctataaGGGCCATGTGATCTTAGTGTCCACG |
|  |  | LRP1_18b | CGTGACGTAGTCCACGGTCAGGCCAaactaccctacaaatccaat |
|  |  | LRP1_19a | ctcactcccaatctctataaTGATGTCCTGGCATCCGGGCTGGCC |
|  |  | LRP1_19b | AGCAGGTACCAAAGCGCAGGCACTCaactaccctacaaatccaat |
|  |  | LRP1_20a | ctcactcccaatctctataaGCAAAATCAGCATCCAGTAGGCCCC |
|  |  | LRP1_20b | AAGTTGGTAGGCTTGTCAGGGTCAAaactaccctacaaatccaat |

| **Gene** | **Amplifier** | **ID** | **Sequence** |
| --- | --- | --- | --- |
| Fgf8 | B3 | FGF8_1a | gtccctgcctctatatctttTGGAGCCGGTGGCCGCTGGCTGCTC |
|  |  | FGF8_1b | GCGCGCCGAACCCCTCTGAGCCGCTTttccactcaactttaacccg |
|  |  | FGF8_2a | gtccctgcctctatatctttGGGACAAGCCGAAGGTGCGGAGGCT |
|  |  | FGF8_2b | TCACGCCGTCCCACTGGAGGCCGCTTttccactcaactttaacccg |
|  |  | FGF8_3a | gtccctgcctctatatctttAGAGAACCAGCAAGTGCAACAGCAG |
|  |  | FGF8_3b | CGCCCGGGCCTTCCTGGGCTTGGATTttccactcaactttaacccg |
|  |  | FGF8_4a | gtccctgcctctatatctttGTGAGGACTGAACAGTTACCTGTTG |
|  |  | FGF8_4b | GCTCCCTCACATGCTGTGTAAAATTTTttccactcaactttaacccg |
|  |  | FGF8_5a | gtccctgcctctatatctttTGGCCAGGACCTGCACGTGCTTCCC |
|  |  | FGF8_5b | CTTCTGCCATGGCGTTGATGCGCTTTTttccactcaactttaacccg |
|  |  | FGF8_6a | gtccctgcctctatatctttTGCAGATGTAGAGACCTGTCTCTGC |
|  |  | FGF8_6b | TGGCAATTAGCTTCCCCTTCTTGTTTTttccactcaactttaacccg |
|  |  | FGF8_7a | gtccctgcctctatatctttACCAGCCCTCGTACTTGGCGTTCTG |
|  |  | FGF8_7b | GCCGGCCCTTGCGGGTAAAGGCCATTTttccactcaactttaacccg |
|  |  | FGF8_8a | gtccctgcctctatatctttGCAGGCTCTGCTCGGTGGTGTGGTG |
|  |  | FGF8_8b | AGGGCGGGTAGTTGAGGAACTCGAATTttccactcaactttaacccg |
|  |  | FGF8_9a | gtccctgcctctatatctttATTCCTCGGCCGGCTGGGTGGGGAG |
|  |  | FGF8_9b | TTTGCTGTGCCGCCGAGCTCCCGCTTTttccactcaactttaacccg |
|  |  | FGF8_10a | gtccctgcctctatatctttCCTCCACCCCAGGGAACCCCAGAGG |
|  |  | FGF8_10b | GGATTTCAGGAGAACAGACCAGAGATTttccactcaactttaacccg |

**
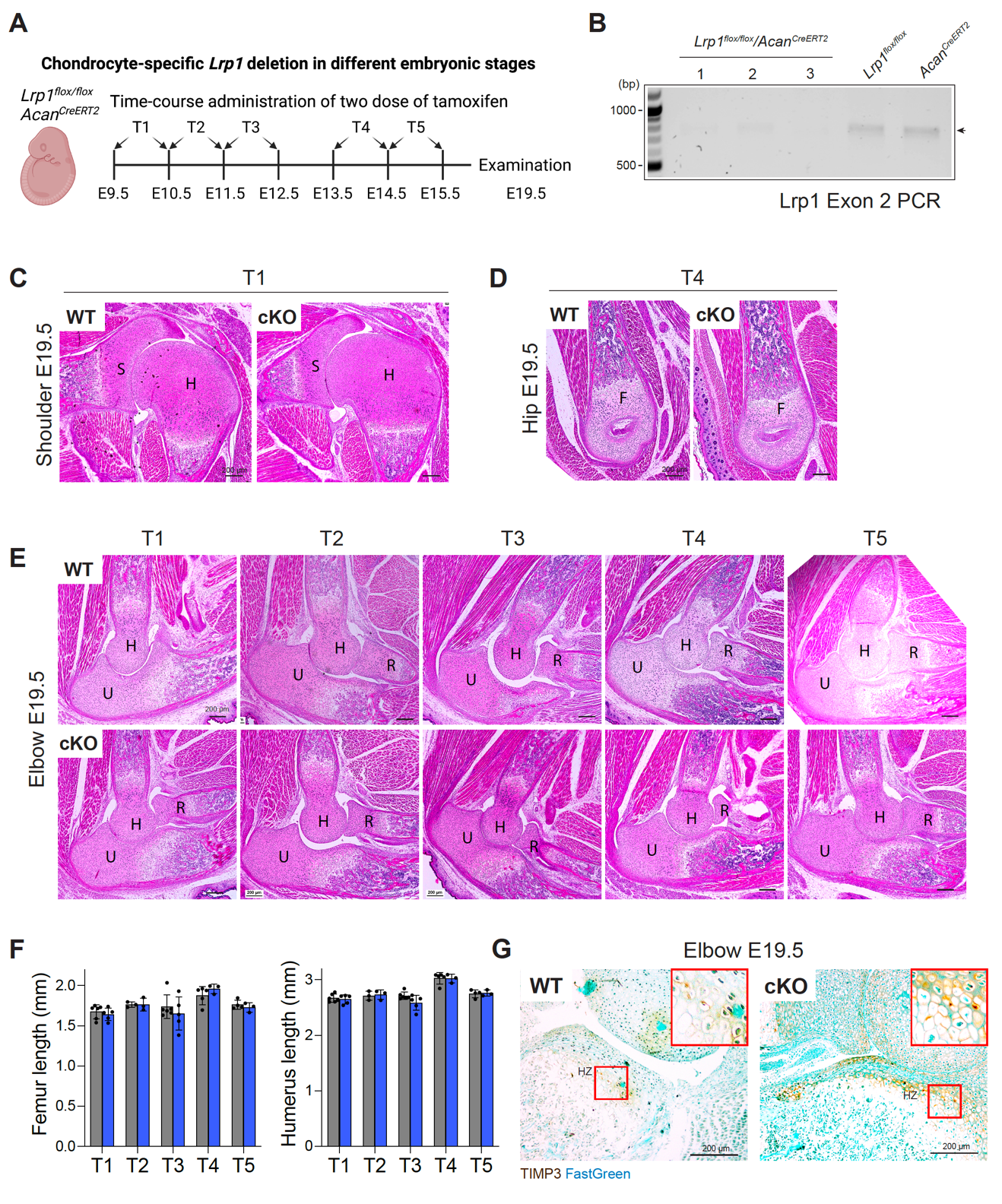
Fig. S1. Conditional deletion of *Lrp1* in chondrocytes does not cause visible abnormality in early bone and joint formation.**

*A*, Schematic diagram showing the chondrocyte-specific *Lrp1* deletion (*Lrp1*^flox/flox^*/Acan*^CreERT2^) in mouse by two doses of tamoxifen gavage administration in the different embryonic stages. *B*, Representative agarose gel electrophoresis for *Lrp1*^flox/flox^*/Acan*^CreERT2^ genotyping to confirm the deletion of *Lrp1* *exon 2* gene. *C-F*, *Lrp1*^flox/flox^*/Acan*^CreERT2^ mice were given two doses of tamoxifen gavage in E9.5 and E10.5 (T1), E10.5 and E11.5 (T2), E11.5 and E12.5 (T3), E13.5 and E14.5 (T4), and E14.5 and E16.5 (T5). Representative images of H&E staining of E19.5 shoulder (*C*), hip (*D*) and elbow (*E*) of WT and cKO mice. Scale bar, 200 µm. S, scapula; H, humerus; U, ulna; R, radius; F, femur. *F*, Femur and humerus bone length of E19.5 WT and *Lrp1*^flox/flox^*/Acan*^CreERT2^ mice. Circles represent individual mice and bars show the mean ± *SD*. *G*, Representative images of immunohistochemical staining of TIMP3 and fast green counterstaining in E19.5 knee sections of WT and *Lrp1*^flox/flox^*/Acan*^CreERT2^ mice, which were given two doses of tamoxifen gavage in E9.5 and E10.5 (cKO). Regions delineated by the red squares have been magnified in the top right of each panel. Scale bar, 200 µm. HZ, hypertrophic zone.

**
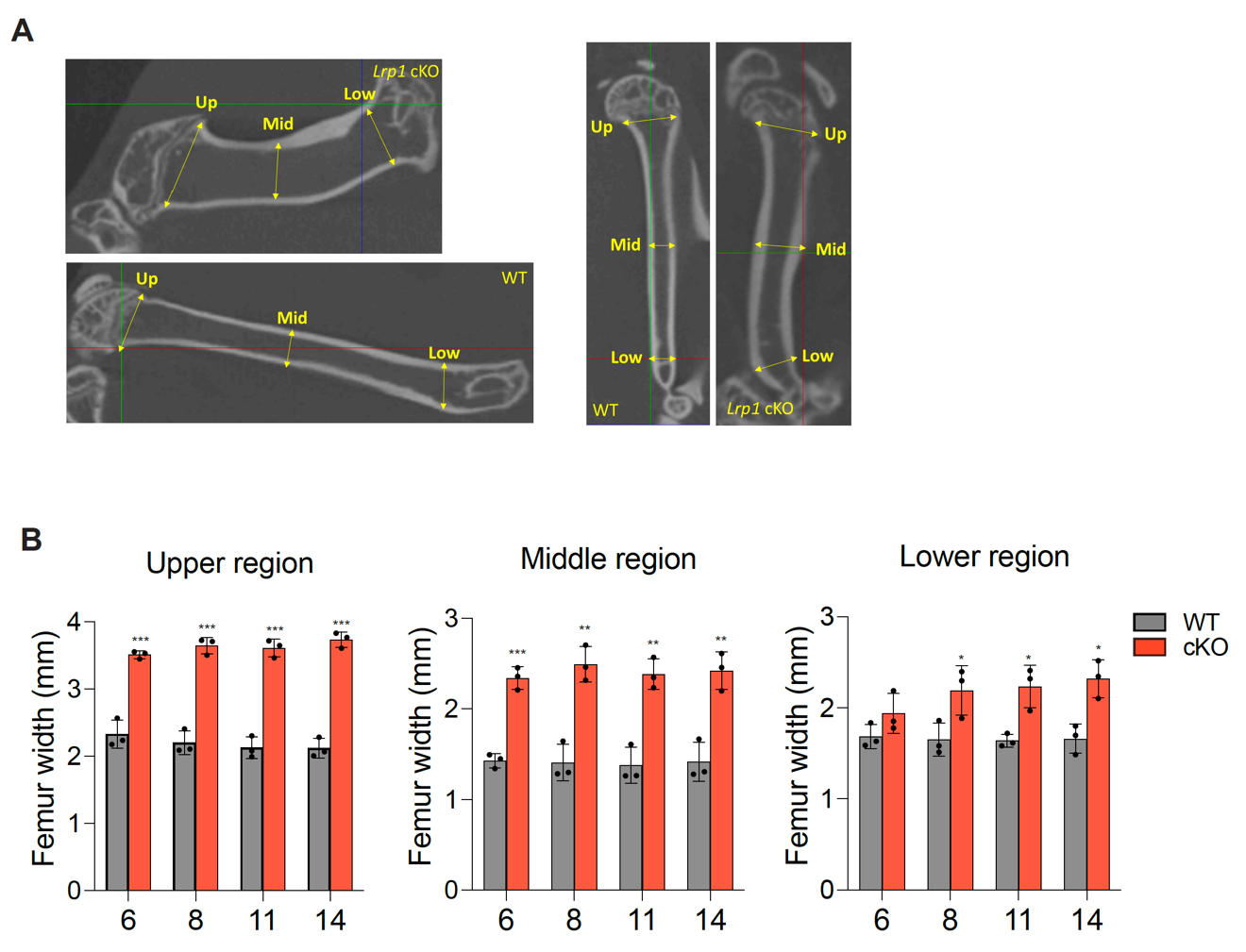
**

**Fig. S2. The upper, middle and lower region of femur width in WT and *Lrp1*^flox/flox^*/Prrx1*^Cre^ mice.**

*A*, Representative images for the bone width measurement on µCT images. The yellow arrows locate where the measurements were taken. (Up: Upper, Mid: Middle, Low: lower). *B*, Femur width at the upper, middle and lower regions of 6-14-week-old mice. Circles represent individual mice and bars show the mean ± *SD*. *, *p* < 0.05; **, *p* < 0.01; ***, *p* < 0.001; ****, *p* < 0.0001 by 2-tailed Student’s t test.

**
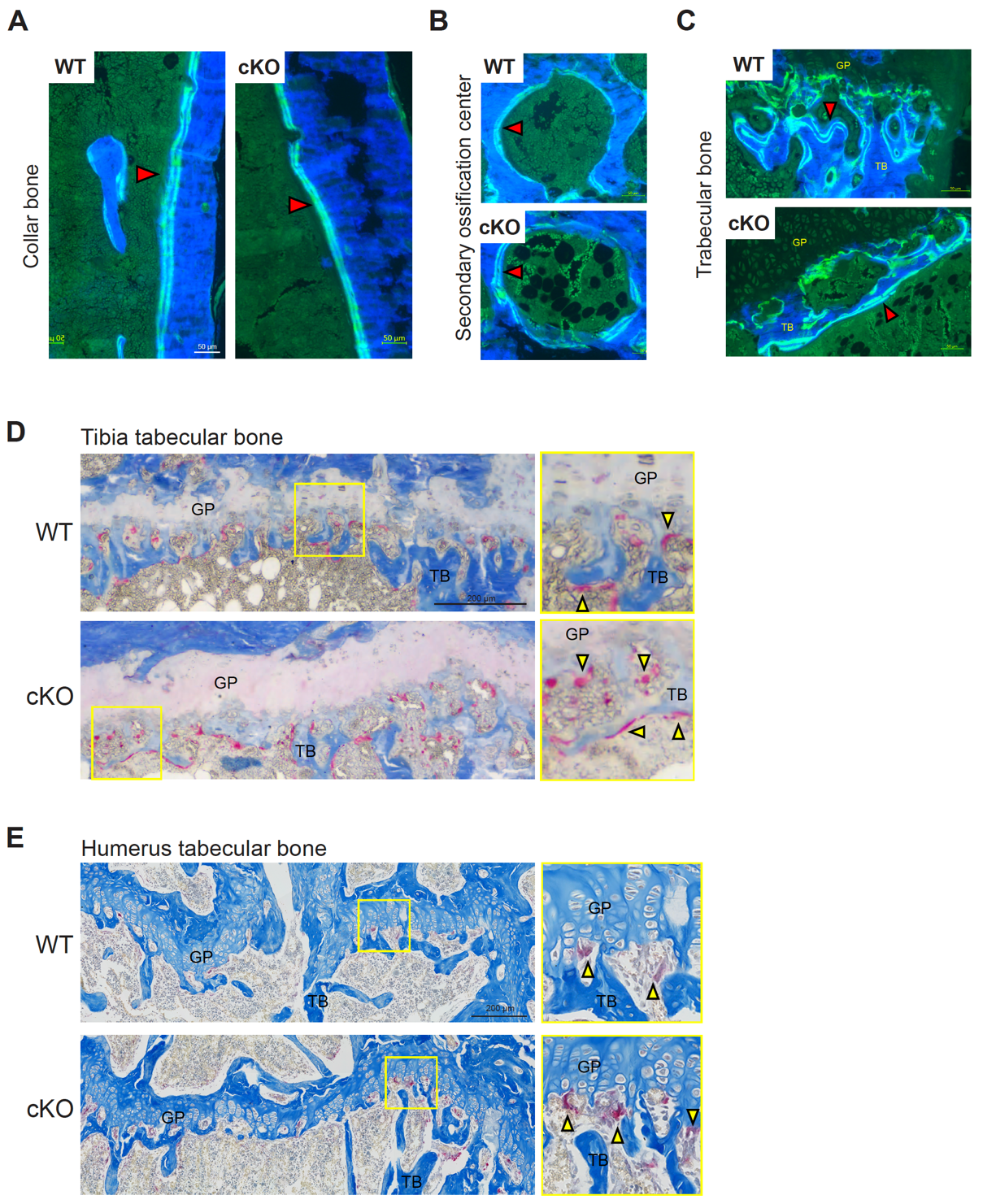
**

**Fig. S3. Comparable bone formation rate and an elevated number of osteoclasts in *Lrp1*^flox/flox^*/Prrx1*^Cre^ compared to WT mice.**

*A*-*C*, Representative images of fluorescent microscopy analysis of calcein-double stained histology sections of the collar bone (*A*), the secondary ossification center (*B*) and the trabecular bone (*C*) of 14-week-old WT and *Lrp1*^flox/flox^*/Prrx1*^Cre^ (cKO) mice. Scale bar, 50 µm. Red arrowheads indicate calcein double staining TB, trabecular bone; GP, growth plate. *D* and *E,* TRAP and aniline blue staining of 14-week-old tibia (*D*) and humerus (*E*) trabecular bones. Regions delineated by the squares in the left panels have been magnified in the right panels. Yellow arrowheads highlight osteoclast staining. Scale bar, 200 µm. TB, trabecular bone; GP, growth plate.

**
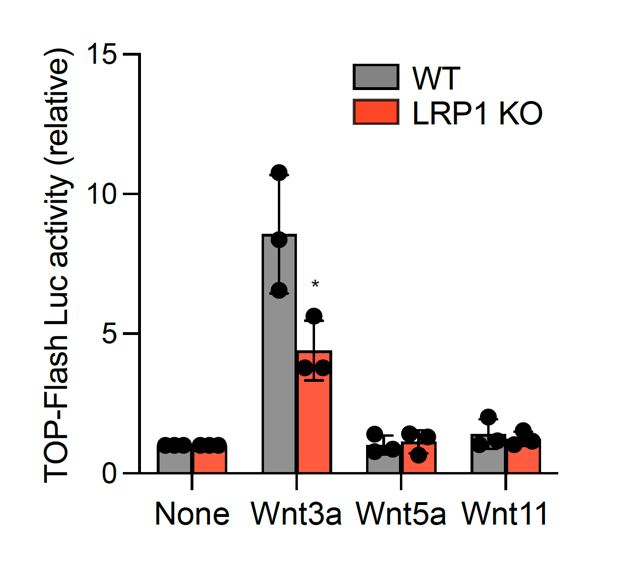
**

**Fig. S4. LRP1 deficiency reduces Wnt3a-induced activation of canonical Wnt singnalling pathway.**

Quantitative Analysis of TOPFlash Luciferase Reporter Assay. WT and LRP1 KO MEFs were co-transfected with either the negative control reporter or the TopFlash reporter, along with the pRL *renilla* luciferase reporter as an internal control. Post-transfection, cells were treated with 100 ng/ml of Wnt3a, Wnt5a, or Wnt11 for 24 h. After 24 h, cells were lysed, and luminescence was measured using a Glomax-Multi luminometer. To normalise the data, TopFlash luminescence values were divided by the pRL luminescence values for each condition. Data shows the relative luciferase activity, Circles represent individual experiment and bars show the mean ± SD. *, *p* < 0.05 by 2-tailed Student’s t test.

**
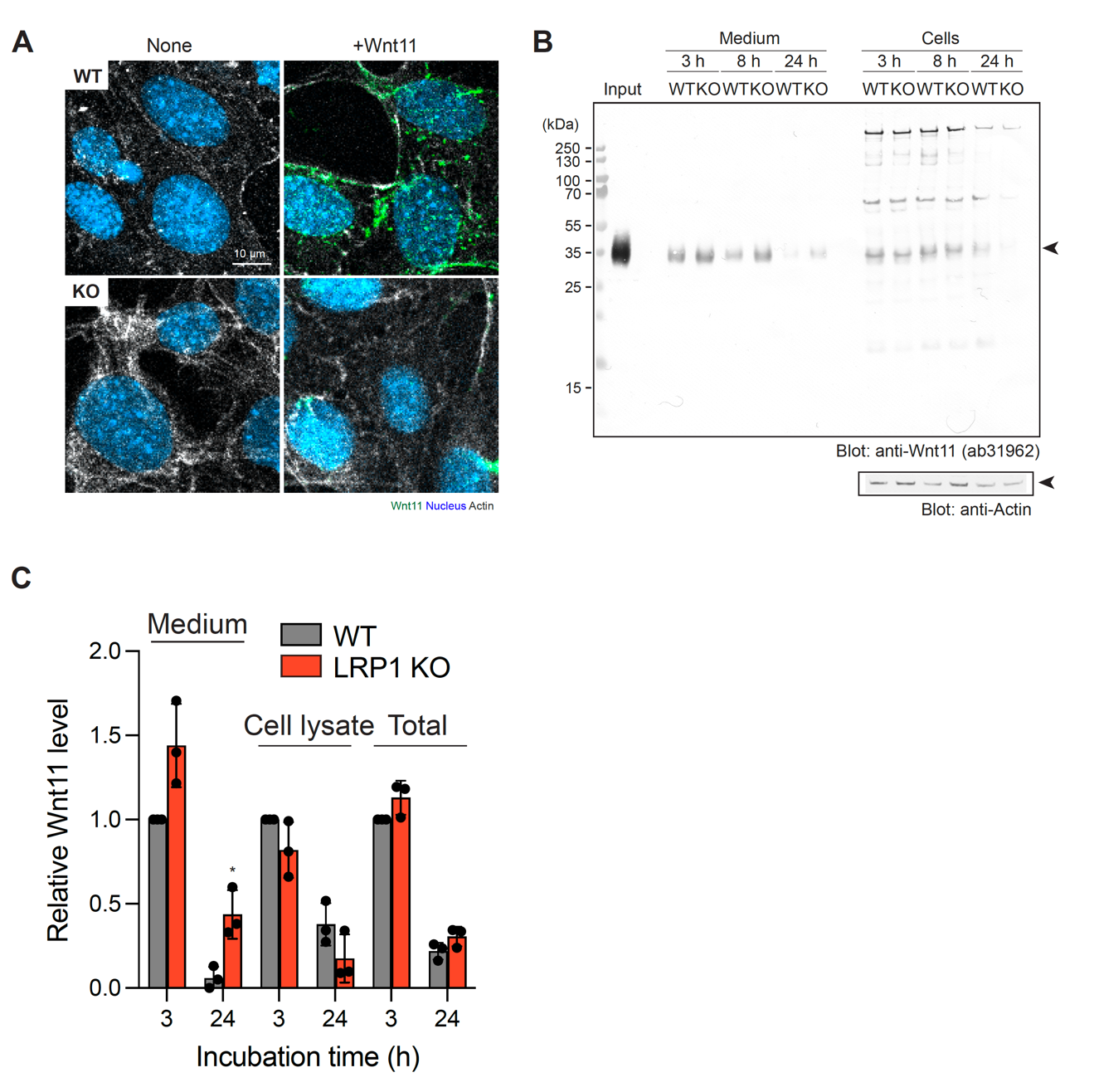
**

**Fig. S5. LRP1-mediated endocytosis of Wnt11.**

*A*, Representative images of confocal microscopy analysis for Wnt11 in WT and LRP1 KO MEFs (n = 3). Cells were incubated with 20 nM Wnt11 for 3 h and Wnt11, cytoskeleton and nucleus were visualised as described under “Supplementary methods”. Scale bar, 10 µm. *B and C*, WT and LRP1 KO MEFs (n = 3) were incubated with 20 nM Wnt11 for 3-24 h and Wnt11 in the medium and cell lysate were detected by Western blotting (*B*). Densitometric analysis of immunoreactive Wnt5a bands was carried out. The relative amount of Wnt11 in the media, cell lysate and both media and cell lysate (total) were expressed by taking the amount of Wn11 after 3-h incubation in WT MEFs as 1 (*C*). Circles represent individual experiment and bars show the mean ± SD. *, *p* < 0.05 by 2-tailed Student’s t test.

**
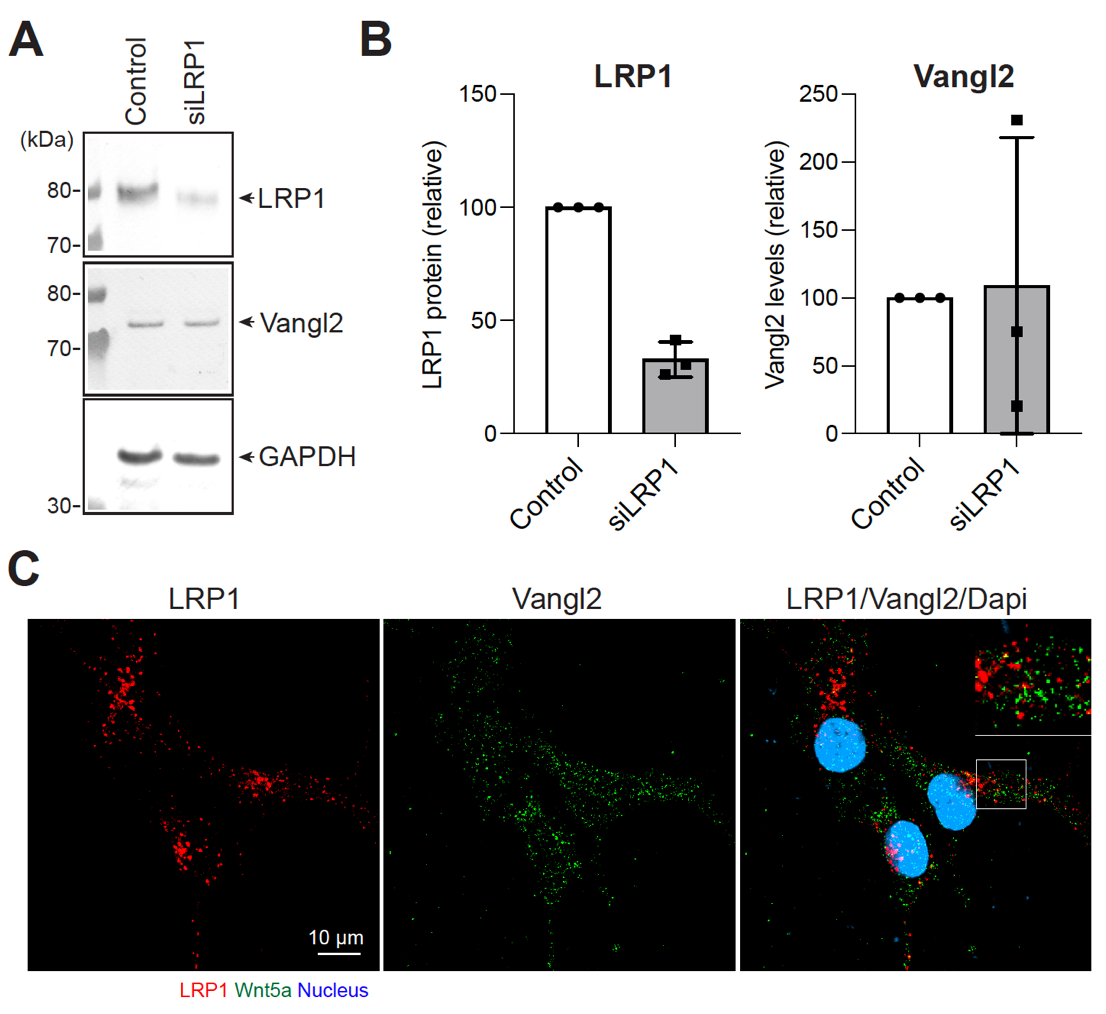
**

**Fig. S6. Effect of siRNA-mediated gene silencing of *Lrp1* on Vangl2 protein levels and subcellular localisation of LRP1 and Vangl2.**

*A* and *B*, Human chondrocytes (n = 3) transfected with non-targeting siRNA (Control) or LRP1 targeting siRNA (siLRP1) were cultured for 2 days in DMEM containing 10% FCS. Representative Western blotting for LRP1 β-chain (85 kDa) and Vangl2 (72 kDa) in cell lysate using anti-LRP1 β-chain (EPR3724) and anti-Vangl2 (sc-515187) antibodies (*A*). Densitometric analysis of immunoreactive LRP1 and Vangl2 bands detected was then carried out and their amount was expressed as a % of their amount in control cells. Circles (control) and squares (siLRP1) represent individual experiment and bars show the mean ± SD. *C*, Representative images of confocal microscopy analysis for LRP1 and Vangl2 in human chondrocytes (n = 3). Cells were incubated with serum-free DMEM for 12 h and LRP1, Vangl2 and nucleus were visualised as described under “Supplementary methods”. Scale bar, 10 µm.

**
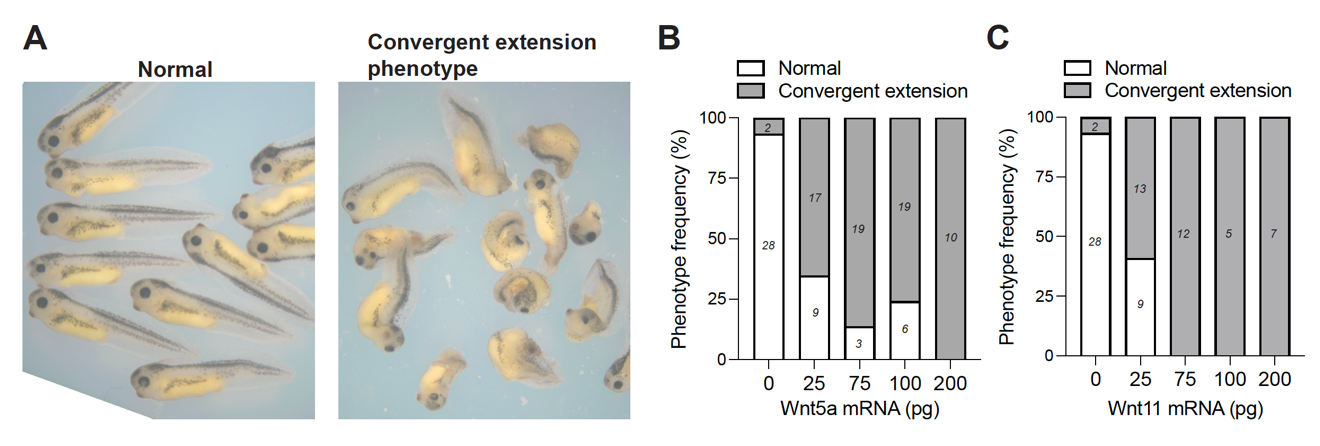
**

**Fig. S7. The WNT/PCP signalling controls convergent extension movements in the developing *Xenopus laevis* embryos.**

*A*, Representative images of normal Xenopus laevis tadpoles and those with disrupted convergent extension after overexpression of Wnt5a mRNA. *B and C*, Various amounts of mRNAs for *Wnt5a* (*B*) or *Wnt11* (*C*) were injected into 1 cell of the dorsal marginal zone of 4-cell stage embryos. Normal embryos and those with a convergent extension phenotype were counted after fixation at stage 35/36.

**
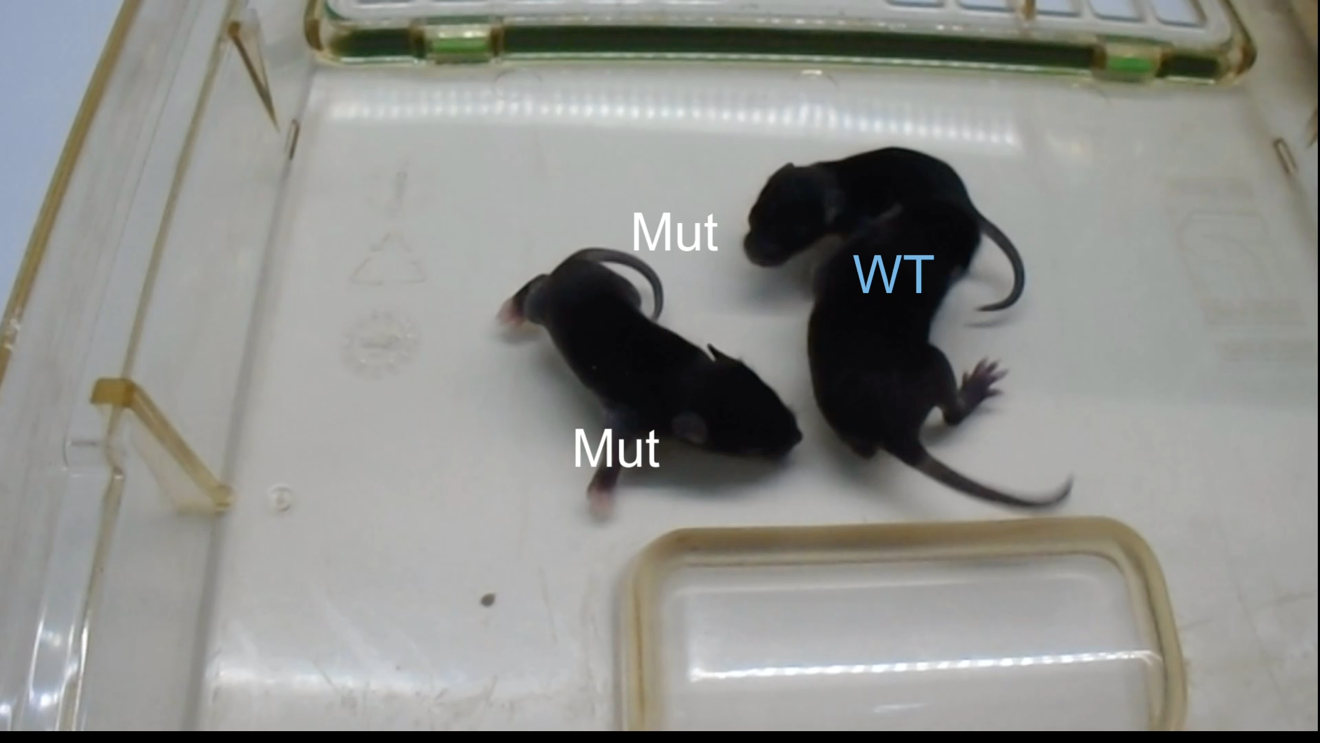
**

**Movie S1. Abnormal “crawling” gait of 2-week-old Lrp1 deficient mice.**

A short video showing abnormal gait of 2-week-old *Lrp1*^flox/flox^*/Prrx1*^Cre^ mice (Mut), which constantly show altered posture and impaired mobility. WT; wild-type littermate.

**
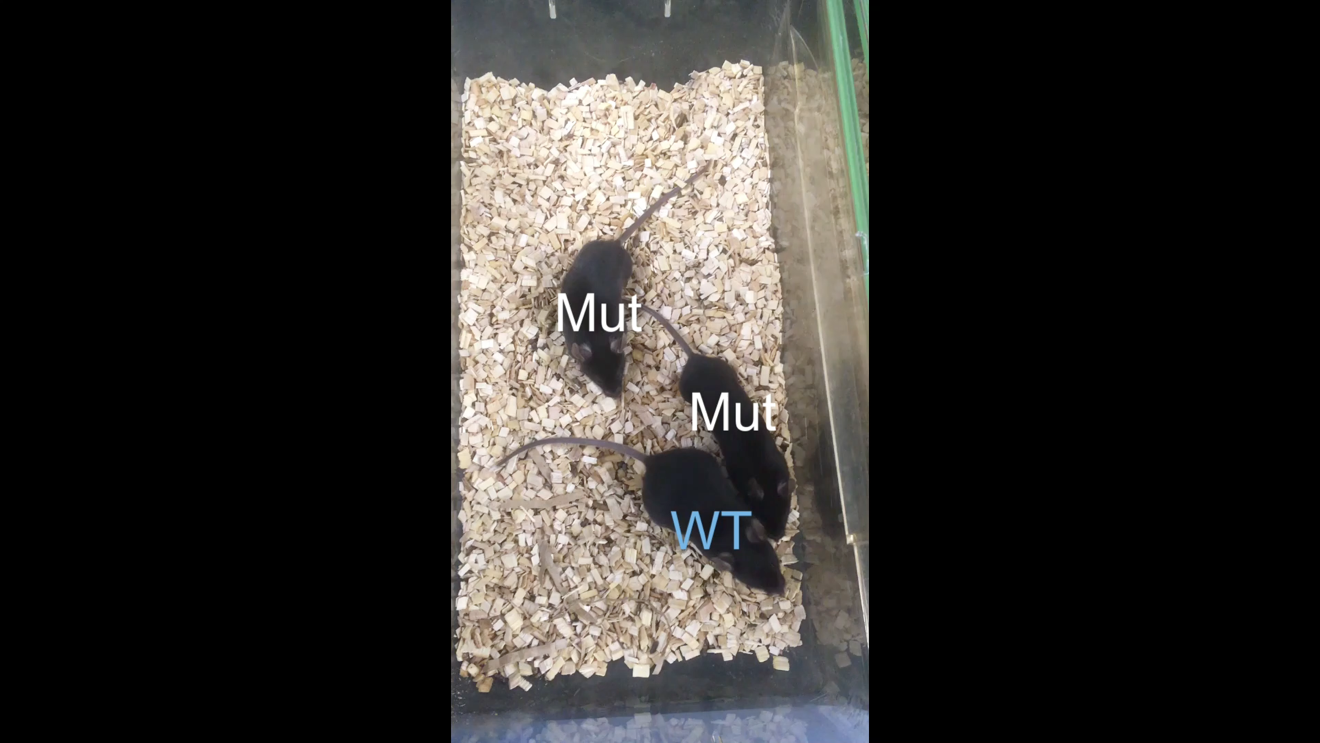
**

**Movie S2. Abnormal “crawling” gait of 13-week-old Lrp1 deficient mice.**

A short video showing abnormal gait of 14-week-old *Lrp1*^flox/flox^*/Prrx1*^Cre^ mice (Mut), which constantly show altered posture and impaired mobility. WT; wild-type littermate.
